# Effect of an integrated intervention on the availability and completeness of Robson ten group classification system-related data in district hospitals in Bangladesh

**DOI:** 10.7189/jogh.16.04069

**Published:** 2026-02-27

**Authors:** Lubna Hossain, Abu Sayed Md Hasan, Hassan Rushekh Mahmood, Farhia Azrin, Anisuddin Ahmed, Abu Sayeed, Sabrina Jabeen, Ema Akter, Haroon Bin Murshid, A K M Mahmudul Hassan, Md. Mahiur Rahman, Trisha Mallick, Tajrin Tahrin Tonmon, Md. Abu Bakkar Siddique, Shamsuz Zaman, Vibhavendra S Rasghuvanshi, Afruna Rahman, Nuzhat Nadia, Mustufa Mahmud, Md Azizul Alim, Ahmed Ehsanur Rahman, Dewan Md Emdadul Hoque, Shams El Arifeen

**Affiliations:** 1 Maternal and Child Health Division, International Centre for Diarrhoeal Disease Research, Bangladesh, Dhaka, Bangladesh; 2 United Nations Population Fund, Dhaka, Bangladesh; 3 Global Health and Migration Unit, Department of Women's and Children's Health, Uppsala University, Uppsala, Sweden; 4 Infectious Diseases Division, International Centre for Diarrhoeal Disease Research, Dhaka, Bangladesh; 5 Maternal, Newborn, Child & Adolescent Health, Directorate General of Health Services, Ministry of Health & Family Welfare of Bangladesh, Dhaka, Bangladesh; 6 United Nations International Children’s Emergency Fund, Dhaka, Bangladesh

## Abstract

**Background:**

The Government of Bangladesh's routine data capturing system of health care facilities doesn’t adequately capture Robson ten group classification system (TGCS)-related variables required to monitor caesarean section rates. To address this gap, we developed an integrated intervention and assessed its impact on the Robson TGCS-related variables availability and completeness.

**Methods:**

We conducted implementation research in eight district hospitals in Bangladesh from January 2021 to June 2023, where the primary population was mothers who gave birth within the implementing health care facilities. Our integrated intervention included the introduction of Robson TGCS; development and implementation of ‘Robson TGCS Report Form’; capacity development training; a multi-level monitoring and supportive supervision system; and collaborative stakeholders’ advisory workshops and review meetings. The primary outcome was the percentage of availability and completeness of TGCS-related data across study phases. The study was divided into five phases. Phase one focused on developing the data-capturing system, while data collection began in Phase two.

**Results:**

The average availability of six variables improved significantly (*P* < 0.001) from 76% (95% confidence interval (CI) = 75.7–76.8%) in phase two to 99% (95% CI = 98.8–99.1%) in Phase five. Similarly, data completeness for these variables increased significantly (*P* < 0.001) from 53% (95% CI = 52.2–54.2%) to 97% (95% CI = 96.2–97%). Consequently, the percentage of classified groups improved significantly (*P* < 0.001) from 55% (95% CI = 54.1–56.1%) to 97% (95% CI = 96.8–97.5%).

**Conclusions:**

The integrated intervention significantly improved data availability and completeness. Active engagement of stakeholders, including governmental bodies and technical experts from local and central levels, is crucial to ensure data quality for identifying, planning and implementing targeted interventions.

In Bangladesh, the rate of caesarean sections (c-sections) has risen sharply in recent years, rising from 41.4% in 2022 to 50.7% in 2023 according to the Bangladesh Sample Vital Statistics 2023 report [1]. This rise is alarming, as in 2018, a study found that 77% of all c-sections in the country were medically unnecessary [2], exposing women and newborns to avoidable surgical risks and longer recovery without proportional health benefits [2–5]. The WHO does not recommend a specific c-section rate at the country level; however, evidence suggests that population-level c-section rates above 10–15% do not confer additional reductions in maternal or neonatal mortality [6,7].

World Health Organization recommends the Robson 10 group classification system (TGCS) as a standardised tool to monitor and compare c-section use across health facilities [8,9]. This classification system categorises pregnant women into 10 mutually exclusive and totally inclusive groups based on key obstetric characteristics: parity, onset of labour, gestational age, foetal presentation, number of foetuses and previous c-section. Together, these six variables provide a simple, objective, and nearly universally available way to describe a woman’s obstetric risk profile [10]. Its successful application depends critically on the systematic availability and completeness of these core variables within routine health information systems [8].

Although the Robson TGCS has been widely adopted and proven effective in various global settings [11–13], its adaptation and consistent implementation in low-resource settings, such as Bangladesh, are constrained by significant data system limitations. Our variable mapping of the existing government reporting system revealed that several essential Robson variables, particularly onset of labour, foetal presentation and previous c-section history, were either absent from the variable list, not captured directly, or recorded inconsistently. These gaps are compounded by high clinical workloads, limited provider training on TGCS, and the absence of the Robson variable and Robson-specific fields in national registers such as the Emergency Obstetric and Newborn Care (EmONC) register. As a result, routine classification of deliveries using the Robson TGCS has not been systematically feasible for monitoring and quality improvement.

To address these system gaps, we developed an integrated, stakeholder-driven intervention to improve the availability and completeness of the Robson TGCS-related variables in eight selected district hospitals (DHs) in Bangladesh. This study aimed to evaluate the effectiveness of this integrated intervention in strengthening Robson TGCS-related data availability and completeness across sequential phases of its implementation.

## METHODS

### Study design and site

We conducted implementation research from January 2021 to June 2023 in eight selected DHs located in Munshiganj, Bogura, Gaibandha, Netrokona, Sunamganj, Bagerhat, Bhola, and Khagrachari from eight divisions of Bangladesh (Figure S1 in the **Online Supplementary Document**). District hospitals serve as referral centres providing secondary-level health care services. These health care facilities were selected based on recommendations from the Maternal, Newborn, Child, and Adolescent Health programme of the Directorate General of Health Services (DGHS), Bangladesh. Additionally, two selection criteria were applied: the facilities needed to provide both vaginal delivery and c-section services and have a team comprising Obstetrics and Gynaecology (ObGyn) consultants and an anaesthesiologist available **(**Table S1 in the **Online Supplementary Document**).

### Study population

In this study, the population was divided into two key categories. The primary population included mothers who gave birth at implementing health care facilities from September 2021 to June 2023. This group was targeted for outcome measurement. The secondary population consisted of frontline service providers, including doctors, nurses, midwives, and facility managers, who play pivotal roles in delivering health care services, including childbirth. The integrated intervention targeted the secondary population (health care providers), while outcomes were assessed among the primary population (mothers) (Figure S2 in the **Online Supplementary Document**).

### Component of integrated intervention

We consulted central and local-level stakeholders from the DGHS (Maternal, Newborn, Child, and Adolescent Health programme), the Obstetrical and Gynaecological Society of Bangladesh (OGSB), United Nations Population Fund (UNFPA), United Nations (UN) agencies, national and international non-governmental organisations, International Centre for Diarrhoeal Disease Research, Bangladesh (icddr,b), district hospital superintendents, Obstetrics and Gynaecology consultants, and Resident Medical Officers, to develop an integrated intervention package (Figure S3 in the **Online Supplementary Document**). The intervention included the introduction of the Robson TGCS; development and implementation of the ‘Robson TGCS Report Form’; capacity development training; a multi-level monitoring and supportive supervision system; and collaborative stakeholder advisory workshops and review meetings to ensure continuous improvement and adherence to data standards. This stakeholder-driven approach ensured contextual relevance, gained ownership, and addressed practical challenges on the ground.

### Implementing the integrated intervention

This study was conducted under the guidance of DGHS with technical support from the OGSB. Following a comprehensive literature review and expert consultations, we developed an integrated intervention plan, which was updated and modified throughout the study period in response to stakeholder recommendations. The study followed a structured approach divided into five phases, each based on data quality findings and stakeholder feedback. This phased approach was designed to focus on key steps in improving data quality, such as identifying data gaps, developing strategies for data collection and management, implementing quality assurance measures, monitoring data accuracy, and continuously adjusting the plan based on stakeholder input. These steps were designed to facilitate gradual learning and adjustments, and ensure effective collaboration with stakeholders (Figure S4 in the **Online Supplementary Document**). The distinct five phases of the Robson TGCS-related data quality improvement process are outlined as follows:

#### Phase 1. Identification (January 2021–July 2021)

The phase focused on the national-level approach, stakeholder engagement, and variable mapping. A comprehensive desk review of routine registers was conducted to identify gaps in Robson TGCS-related variables. A total of 17 national- and 40 district-level stakeholders, including representatives from government health programmes, professional societies, UN agencies, national and international non-governmental organisations, icddr,b, and health service providers from the study facilities, were identified during this phase. Study sites were finalised in consultation with DGHS and OGSB.

#### Phase 2. Sensitisation (August 2021–April 2022)

National and district stakeholders were sensitised on the importance of Robson TGCS. The ‘Robson TGCS Report Form’ was developed, finalised and endorsed. Healthcare providers were trained virtually on Robson TGCS and standardised documentation due to COVID-19 restrictions. Routine monthly data collection commenced during this phase, alongside district-level monitoring by DGHS-appointed Sexual and Reproductive Health and Rights officers, who were DGHS-recruited technical staff at the district level, with funding support from UNFPA.

#### Phase 3. Involvement (May 2022–July 2022)

In the third phase, face-to-face training sessions on the ‘Robson TGCS’, ‘EmONC Register’ and ‘Robson TGCS Report Form’ were provided. A structured multi-tier monitoring and supportive supervision system was implemented involving facility staff, district Sexual and Reproductive Health and Rights officers and the icddr,b research team.

#### Phase 4. Engagement (August 2022 –December 2022)

Central-level stakeholders from DGHS and OGSB were engaged in joint supportive supervision with district teams. Refresher training and structured review meetings were conducted to address emerging data quality gaps.

#### Phase 5. Engagement (January 2023–June 2023)

All core components of the integrated intervention, including routine data recording, monitoring, and supportive supervision, were continued during this phase. In addition, need-based refresher training was provided at selected hospitals to address staff turnover rather than regular refresher training. Dissemination workshops were conducted to share findings with national- and district-level stakeholders.

The intervention required inputs for provider training, routine supervisory and supportive monitoring, structured stakeholder consultations at central and district levels, and dedicated data management personnel for data extraction, validation, entry, and quality assurance (Figure S5 in the **Online Supplementary Document**).

### Data collection process

The research team adopted a systematic approach to collecting data on Robson TGCS from the second phase. Doctors, nurses, and midwives were responsible for capturing data, specifically who conducted the delivery, in both the EmONC Register and the Robson TGCS Report Form. Field Research Officers extracted data monthly from the ‘EmONC Register’ and the ‘Robson TGCS Report Form’ after validation by ObGyn consultants or relevant personnel at the study site. Extracted data was then entered into icddr,b’s in-house Data Management System by the Data Management Assistant. After completing data entry, the Project Physician and statistician from the research team conducted a rigorous review to ensure the quality and accuracy of the entered data.

### Variables

#### Covariates

We explored the background characteristics of mothers who delivered in the facilities within the given time frame. The variables we were considered were age (<20 years, 20–34 years, >34 years), gestational week (<28 weeks, ≥28 weeks), gravida (one, two or more), received antenatal care (Yes, No), partograph used (Yes, No), pregnancy outcome (live birth, intrauterine death (IUD), stillbirth), sex of the newborn (male, female), weight of the baby (<2.5 kg, 2.5–4.5 kg, >4.5 kg), postnatal care (first, second), facility name (Munshiganj DH, Bogura DH, Gaibandha DH, Netrokona DH, Sunamganj DH, Bagerhat DH, Bhola DH, Khagrachari DH), postpartum family plan method (Yes, No), mode of delivery (vaginal delivery, c-section). Covariates were selected *a priori* based on clinical relevance and availability in routine facility registers, as supported by the literature.

#### Outcome variables

Robson TGCS applies to pregnancies ≥28 weeks’ gestation; therefore, records with gestational age <28 weeks were excluded from outcome analyses. We assessed data quality based on variable availability and completeness across different study phases. Availability was defined as the presence of each individual Robson TGCS-related variable in the register. For each variable, data were categorised as ‘available’ if the variable was recorded for a given mother, and ‘not available’ if the variable was not recorded. Additionally, completeness was defined as the presence of all six Robson TGCS-related variables for each mother. For each mother, data were categorised as ‘complete’ if all six variables were recorded, and ‘not complete’ if any one or more of the six variables were not recorded. To ensure a thorough assessment, cases with unavailable gestational age were included in the analysis. This evaluation allowed us to determine the classified and unclassified percentages of Robson TGCS, which is another component to monitor data quality. In the context of Robson TGCS, the classified group includes mothers who have predefined variables required for Robson classification, while the unclassified group consists of mothers with the unavailability of one or more variables due to incomplete information. Additionally, we also calculated the relative contribution of the c-section to the unclassified group to explore the change and data quality improvement in contribution across phases.

Availability = a × 100 / n

where ‘a’ is the number of mothers with the presence of a specific variable and ‘n’ is the total number of mothers considered.

Completeness = c × 100 / n

where ‘c’ is the number of mothers with all six variables present, and ‘n’ is the total number of mothers considered.

### Data analysis plan

A comprehensive descriptive analysis was conducted using the statistical software STATA 15 (StataCorp LLC, College Station, Texas, USA). Additionally, by adjusting phase (two, three, four, five), facility name (Munshiganj DH, Bogura DH, Gaibandha DH, Netrokona DH, Sunamganj DH, Bagerhat DH, Bhola DH, Khagrachari DH), mode of delivery (vaginal delivery, c-section) and pregnancy outcome (live birth, IUD, stillbirth), a logistic regression was conducted to capture the effect of these components on data completeness. For this regression analysis, we categorised completeness into two categories, referring to the presence of all six variables named as ‘completeness’ and the absence of any variable indicates ‘not completed’. To account for facility-level variability and temporal changes, models were adjusted for facility and study phase. Robust standard errors clustered at the facility level were applied to account for within-facility correlation. Adjusted odds ratios (ORs) with 95% confidence intervals (CIs) were reported.

## RESULTS

A total of 29 634 women delivered in between Phases two and five in all the eight DHs with an average of 1347 births per month. The majority of women were in the age group 20–34 years (80%); 58% of them were multigravida and 88% of them received at least one antenatal care visit. Overall, 77% of deliveries were vaginal delivery, and 23% were by c-section (**Table 1**). Out of all the phases, Sunamganj DH and Netrokona DH together contributed over one-third (35%) of total deliveries in every phase.

**Table 1:** Background characteristics by phases in eight DHs in Bangladesh

Background characteristics	Phase two (September 2021–April 2022), N (%)	Phase three (May 2022–July 2022), N (%)	Phase four (August 2022–December 2022), N (%)	Phase five (January 2023–June 2023), N (%)	Total (September 2021–June 2023), N (%)
Age (year)					
*<20*	1168 (12)	489 (13)	1136 (14)	925 (12)	3718 (13)
*20–34*	8042 (81)	2928 (80)	6528 (79)	6286 (81)	23 784 (80)
*>34*	677 (7)	249 (7)	600 (7)	528 (7)	2054 (7)
*Missing*	68 (1)	5 (0)	4 (0)	1 (0)	78 (0)
Gravida					
*1*	4131 (41)	1536 (42)	3423 (41)	3047 (39)	12 137 (41)
*≥2*	5668 (57)	2110 (57)	4831 (58)	4671 (60)	17 280 (58)
*Missing*	156 (2)	25 (1)	14 (0)	22 (0)	217 (1)
Gestational week					
*≤27*	296 (3)	174 (5)	186 (2)	130 (2)	786 (3)
*≥28*	8787 (88)	3414 (93)	8050 (97)	7599 (98)	27 850 (94)
*Missing*	872 (9)	83 (2)	32 (0)	11 (0)	998 (3)
Received ANC					
*Yes (At least one)*	8209 (83)	3187 (87)	7451 (90)	7174 (93)	26 021 (88)
*No*	1705 (17)	484 (13)	817 (10)	566 (7)	3572 (12)
*Missing*	41 (0)	0 (0)	0 (0)	0 (0)	41 (0)
Partograph used					
*Yes*	6623 (67)	2445 (67)	5832 (71)	5358 (69)	20 258 (68)
*No*	3291 (33)	1226 (33)	2436 (29)	2382 (31)	9335 (32)
*Missing*	41 (0)	0 (0)	0 (0)	0 (0)	41 (0)
Mode of delivery					
*VD*	7872 (79)	2858 (78)	6354 (77)	5653 (73)	22 737 (77)
*C-section*	2083 (21)	813 (22)	1914 (23)	2087 (27)	6897 (23)
Pregnancy outcome					
*Live birth*	7416(74)	3215 (88)	7732 (94)	7316 (95)	25 679 (87)
*IUD*	472(5)	131 (4)	390 (5)	329 (4)	1322 (4)
*Still birth*	24(0)	4 (0)	7 (0)	6 (0)	41 (0)
*Missing*	2043(21)	321 (9)	139 (2)	89 (1)	2592 (9)
Sex of the newborn					
*Male*	4149 (42)	1795 (49)	4196 (51)	3892 (50)	14 032 (47)
*Female*	3991 (40)	1541 (42)	3824 (46)	3665 (47)	13 021 (44)
*Missing*	1815 (18)	335 (9)	248 (3)	183 (2)	2581 (9)
Weight of the baby (kg)					
*<2.5*	677 (7)	296 (8)	756 (9)	657 (8)	2386 (8)
*2.5–4.5*	7252 (73)	2987 (81)	7126 (86)	6793 (88)	24 158 (82)
*>4.5*	40 (0)	15 (0)	34 (0)	40 (1)	129 (0)
*Missing*	1986 (20)	373 (10)	352 (4)	250 (3)	2961 (10)
PNC					
*1*	4817 (48)	2515 (69)	5394 (65)	4948 (64)	17 674 (60)
*2*	2161 (22)	655 (18)	2298 (28)	2481 (32)	7595 (26)
*Missing*	2977 (30)	501 (14)	576 (7)	311 (4)	4365 (15)
Postpartum family plan method					
*Yes*	3028 (30)	2199 (60)	6395 (77)	5542 (72)	17 164 (58)
*No*	6886 (69)	1472 (40)	1873 (23)	2198 (28)	12 429 (42)
*Missing*	41 (1)	0 (0)	0 (0)	0 (0)	41 (0)
Place of facility					
*Munshiganj DH*	687 (7)	254 (7)	557 (7)	515 (7)	2013 (7)
*Bogura DH*	1249 (13)	372 (10)	921 (11)	982 (13)	3524 (12)
*Gaibandha DH*	810 (8)	256 (7)	561 (7)	511 (7)	2138 (7)
*Netrokona DH*	2112 (21)	582 (16)	1515 (18)	1513 (20)	5722 (19)
*Sunamganj DH*	1820 (18)	703 (19)	1559 (19)	1431 (18)	5513 (19)
*Bagerhat DH*	517 (5)	274 (7)	780 (9)	707 (9)	2278 (8)
*Bhola DH*	1203 (12)	578 (16)	1065 (13)	890 (11)	3736 (13)
*Khagrachari DH*	1557 (16)	652 (18)	1310 (16)	1191 (15)	4710 (16)
*Total*	9955 (100)	3671 (100)	8268 (100)	7740 (100)	29 634 (100)

ANC – antenatal care, C-section – caesarean section, DH – district hospital, PNC – postnatal care, VD – vaginal delivery, IUD– intrauterine death

**Figure 1:**
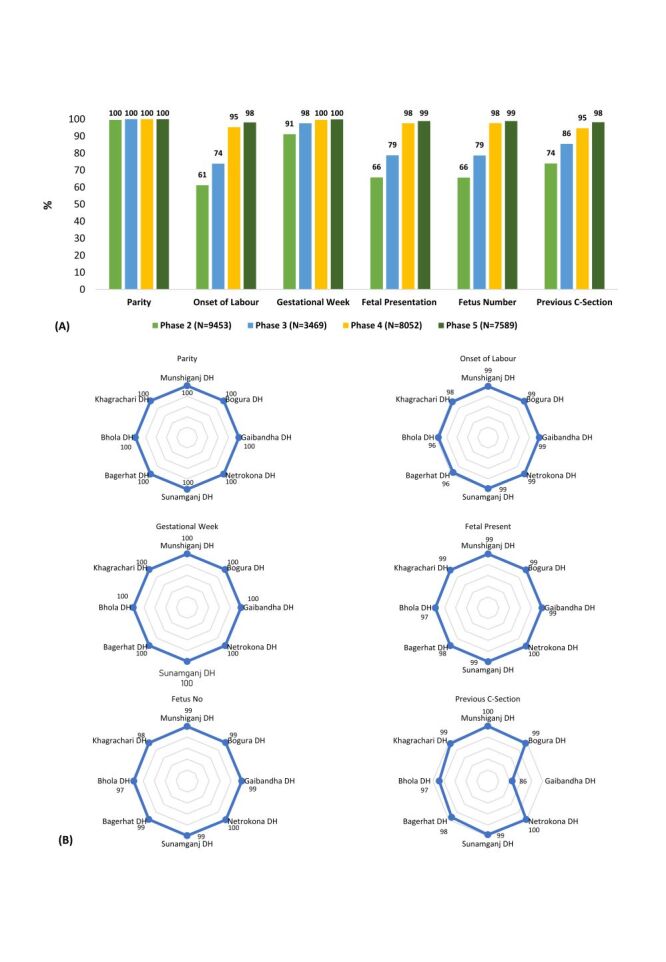
**Panel A**. Phase-wise availability (%) of Robson TGCS-related variables. **Panel B**. Facility-wise availability (%) of Robson TGCS-related variables in Phase five across eight district hospitals in Bangladesh. TGCS – ten group classification system.

Over the period of the study, there was a significant improvement (*P* < 0.001) in the documentation of all the Robson TGCS variables (**Figure 1**, Panel A). The average availability of variables significantly improved from 76% (95% CI = 75.7–76.8%) in Phase two to 99% (95% CI = 98.8–99.1%) in Phase five (Table S2 in the **Online Supplementary Document**). As depicted from the **Figure 1**, nearly all variables demonstrated notable improvements. The onset of labour data availability increased from 61% (95% CI = 60.2–62.2%) in Phase two to 98% (95% CI = 97.8–98.4%) in Phase five. Similarly, foetal presentation and number of foetuses consistently increased across the phases, with 66% (95% CI = 64.9–66.8%) in Phase two to 99% (95% CI = 98.6–99.1%) in Phase five (**Figure 1**, Panel A; Table S2 in the **Online Supplementary Document**), while previous c-section history improved from 74% (95% CI = 73.1–74.9%) in Phase two to 98% (95% CI = 97.9–98.5%) in Phase five. Facility-level analyses indicated that nearly all DHs experienced statistically significant improvements (*P* < 0.001), confirming the broad impact of the intervention (Figure S6–13 and Table S3–10 in the **Online Supplementary Document**).

**Figure 2:**
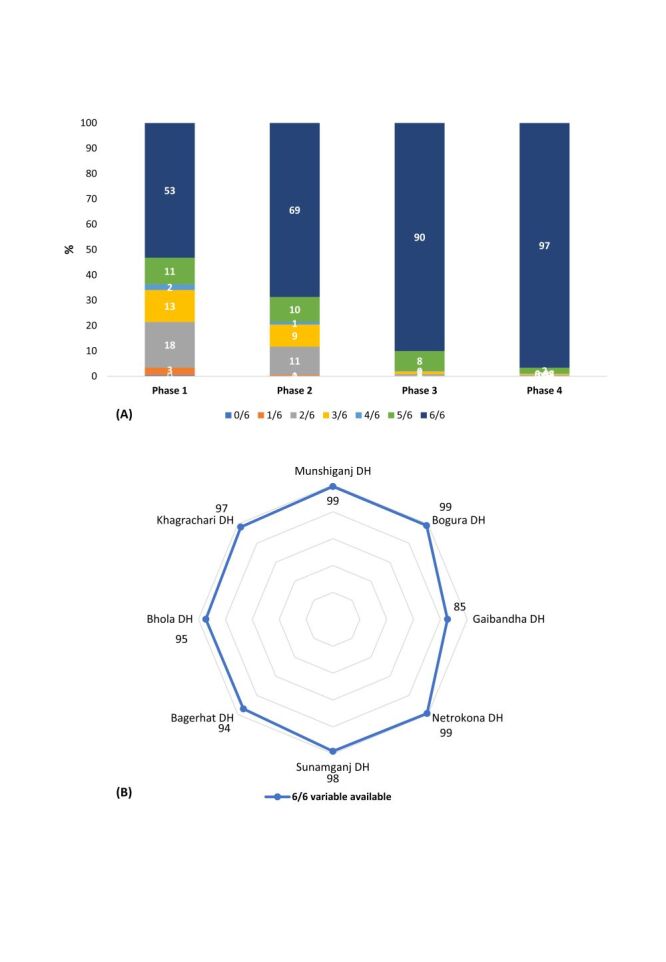
**Panel A**. Phase-wise completeness (%) of six Robson TGCS-related variables. **Panel B**. Facility-wise completeness (%) of six Robson TGCS-related variables in Phase five across eight district hospitals in Bangladesh. TGCS – ten group classification system.

**Figure 2** shows the changes in all six-variable completeness by phase and facility (**Figure 2**, Panels A and B). The overall findings revealed that the variable completeness increased from Phase two to Phase five, with the highest proportion (*P* < 0.001) of having all six variables available in Phase five (96.6%; 95% CI = 96.2–97%) (**Figure 2**, Panel A; Table S11 in the **Online Supplementary Document**). Gaibandha DH had a decline in variable completeness from Phase four, 99% (95% CI = 97.8–99.6%) to 85% (95% CI = 81.8–88.1%) in Phase five (**Figure 2**, Panel B; Figures S14–21 and Tables S12–19 in the **Online Supplementary Document**). Sunamganj, and Bogura DH had the lowest proportion of complete variables in Phase two respectively (25.6%; 95% CI = 23.6–27.7%) and (26.3%; 95% CI = 23.8–28.8%) among all the facilities, which significantly improved to 98.2% (95% CI = 97.3–98.7%) and 98.6% (95% CI = 97.6–99.1%) by Phase five respectively (Figure S14–21 and Table S12–19 in the **Online Supplementary Document**). Additionally, phase-wise adjusted ORs revealed significantly increased odds of completeness compared to Phase two, the odds of completeness were significantly two times higher in Phase three (OR = 2.0; 95% CI = 1.9–2.2), nearly nine times higher in Phase four (OR = 8.9; 95% CI = 8.1–9.7), and in Phase five, participants exhibited approximately 29 times higher odds of completeness (OR = 29.4; 95% CI = 25.6–33.7) than Phase two (Table S20 in the **Online Supplementary Document**). Compared with vaginal delivery, c-section was linked to significantly 28% lower odds (OR = 0.72; 95% CI = 0.65–0.78). In the facilities variation, Bogura DH (OR = 0.40; 95% CI = 0.34–0.48), Sunamganj DH (OR = 0.34; 95% CI = 0.29–0.40), Bhola DH (OR = 0.79; 95% CI = 0.67–0.93), and Khagrachari DH (OR = 0.51; 95% CI = 0.44–0.60) showed significantly lower odds of completeness compared to Munshiganj DH, while higher odds of completeness were found in Gaibandha DH (OR = 2.20; 95% CI = 1.80–2.69, *P* < 0.01) and Netrokona DH (OR = 1.25; 95% CI = 1.06–1.46, *P* = 0.01). Completeness was also lower for adverse pregnancy outcomes, where intrauterine foetal deaths had 34% lower odds (OR = 0.66; 95% CI = 0.56–0.77), and stillbirths had 56% lower odds of the completeness (OR = 0.44; 95% CI = 0.21–0.96) compared to live births (Table S20 in the **Online Supplementary Document**).

**Figure 3:**
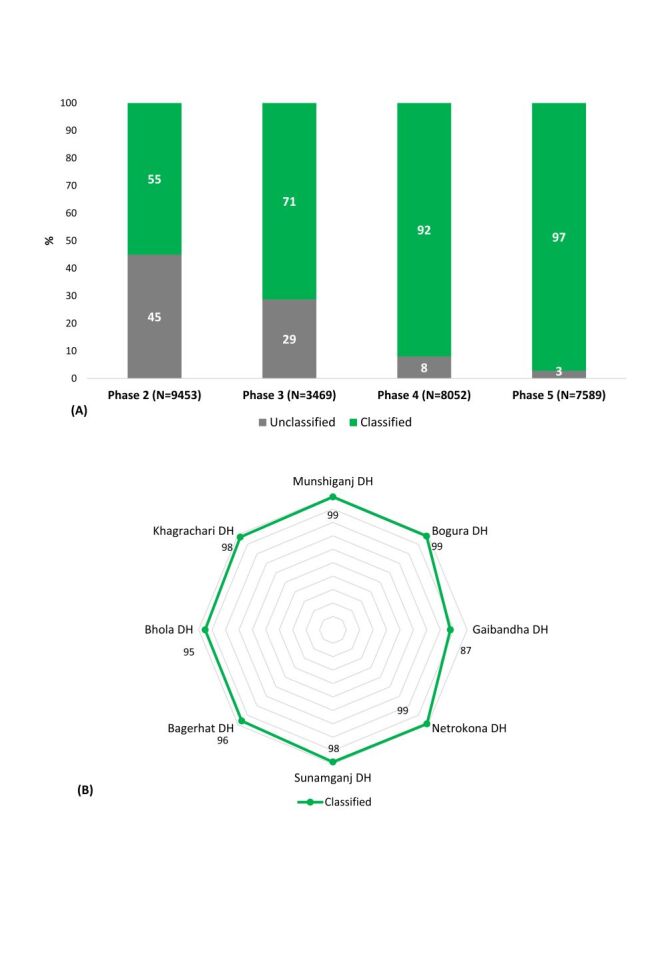
**Panel A**. Phase-wise distribution (%) of classified and unclassified Robson TGCS groups. **Panel B**. Facility-wise distribution (%) of classified Robson TGCS groups in Phase five across eight district hospitals in Bangladesh. TGCS – ten group classification system.

Improvements in both availability and completeness resulted in a significant improvement in the percentage of classified groups from Phase two to Phase five (*P* < 0.001), with the percentage of classified groups increasing from 55% (95% CI = 54.1–56.1%) to 97% (95% CI = 96.8–97.5%) (**Figure 3**; Table S21 in the **Online Supplementary Document**). At the facility level, the lowest percentage of the classified group were observed in Sunamganj DH, both in Phase two (29%; 95% CI = 26.5–30.8%) and Phase three (38%; 95% CI = 33.8–41.6%), while in Phase five the rise of classification rate was 98% (95% CI = 97.6–99.0%) (Figure S22 and Table S21–29 in the **Online Supplementary Document**). However, Gaibandha DH had the lowest percentage of classified Group in Phase five among all the DHs (87%; 95% CI = 84.2–90.3%) (Figure S22 and Table S22–29 in the **Online Supplementary Document**).

**Figure 4:**
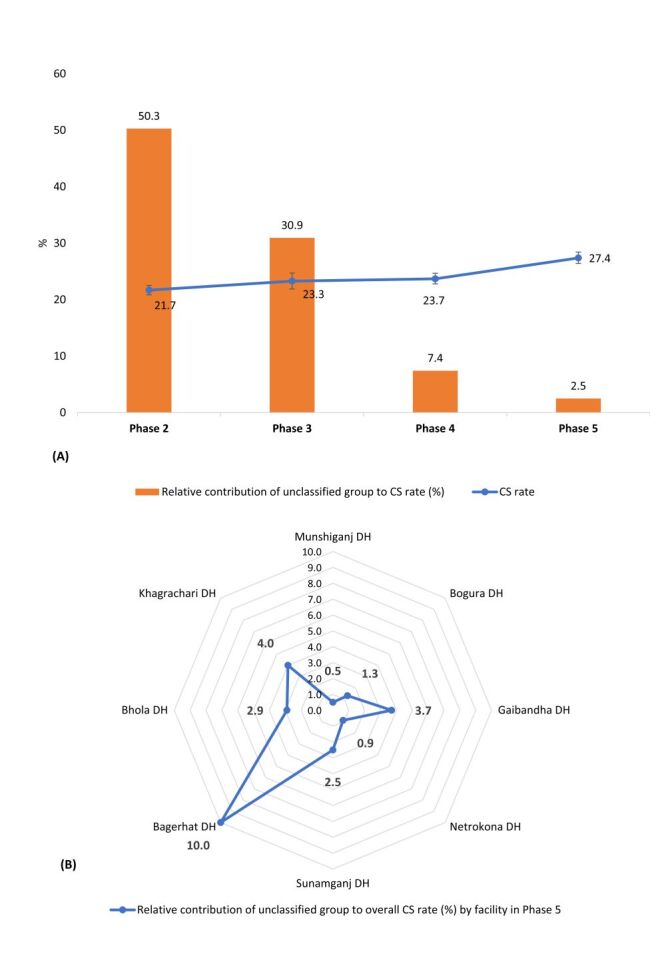
**Panel A**. Phase-wise caesarean-section rate (%) and relative contribution (%) of the unclassified group. **Panel B**. Facility-wise relative contribution (%) of the unclassified group to the overall caesarean-section rate in Phase five.

**Figure 4** represents the change in the relative contribution of the unclassified group to the overall rates of c-sections across phases. At the beginning of the study, the unclassified group was 50% of all c-sections and by Phase five, it had decreased to 3% (**Figure 4**; Table S30 in the **Online Supplementary Document**). In the initial phase, among all of the facilities Bogura, Bhola, and Sunamganj DHs showed the highest relative contribution of unclassified cases to the overall c-section, accounting for 62.9, 74.6, and 80.4%, respectively. In the Phase five, these three facilities showed significant improvements with the relative contribution of unclassified cases dropping to 1.3, 2.9, and 2.5%, respectively (Table S30–38 in the **Online Supplementary Document**). Conversely, Bagerhat DH reported the highest proportion of unclassified cases in Phase five (10%); however, this still reflects a notable improvement compared with the 26.1% recorded in Phase four (Table S31–38 in the **Online Supplementary Document**).

## DISCUSSION

This study demonstrated that an integrated, stakeholder-engaged intervention substantially improved the availability, completeness, and usability of Robson TGCS-related variables in secondary-level public hospitals in Bangladesh. Strengthened routine documentation enabled near-complete Robson classification by Phase five, allowing reliable monitoring of group-specific c-section patterns.

Although improvements closely followed the implementation of the integrated intervention, concurrent health system strengthening activities, increased managerial oversight, and heightened institutional accountability during the study period may also have contributed to the observed gains in documentation. These contextual influences should be considered when interpreting the magnitude of intervention effects.

Near-perfect classified data allowed us to observe accurate distribution across the 10 groups. Our findings regarding the relative sizes of obstetric groups were mostly within the expected ranges. Specifically, groups one and two (35–42%) and groups three and four (30–40%) collectively aligned with existing literature [8]. These estimates were reflected in our overall results, although some variation was noted among individual facilities. Group five, typically representing about half of the total c-section rate [8], aligned with our cumulative findings after data quality improvements in Phase five, despite initial discrepancies. Similar discrepancies were also found for groups one, two, three and four. These discrepancies for groups one through five emphasise the importance of accurate data collection and classification in health care decision-making. The integrated intervention approach demonstrated potential for use in other secondary-level facilities in Bangladesh and similar settings, facilitating targeted interventions to optimise c-sections.

At baseline, major challenges included incomplete routine registers, limited staffing, and low familiarity with Robson TGCS among providers, similar to findings from Myanmar and other low-resource settings [14–16]. The combined use of structured training, stakeholder engagement, and routine supervision successfully addressed these barriers and strengthened routine documentation. Data accuracy was further reinforced through regular verification of register and form entries by ObGyn consultants or their designated personnel at the facility level. Repeated training, feedback, and supervision gradually improved providers' knowledge, confidence, and perceived usefulness of the Robson TGCS, thereby strengthening motivation for accurate and consistent documentation in later phases.

Specific facility-level challenges require need-based responses. Temporary declines in data quality at Bhola and Gaibandha DHs were likely related to staff turnover and nursing shortages. Targeted refresher training and strengthened supervision helped restore documentation performance. The most noticeable improvements in data availability and completeness occurred between Phases three and four and were largely sustained through Phase five, suggesting that the institutionalisation of intensive monitoring, face-to-face refresher training, supportive supervision, and central-level stakeholder engagement contributed to a stable and sustained level of data quality. Our findings underscore that active engagement by central and local-level stakeholders is critical to sustaining data quality during Robson TGCS implementation. Collaborative supervision involving clinicians, managers, and policymakers enabled context-appropriate solutions and strengthened institutional ownership, which is essential for long-term sustainability [17,18]. Transitioning to electronic or hybrid (paper-electronic) reporting systems could further enhance data completeness, reduce transcription errors, and enable real-time monitoring. However, feasibility in low-resource settings depends on stable electricity, internet connectivity, hardware maintenance, and provider digital literacy, without which long-term sustainability may be compromised [19–28].

Institutionalising the Robson TGCS variables within the national health information system is essential for routine monitoring of c-section practices. As the existing government data-capturing system in Bangladesh is primarily paper-based, IT infrastructure was not a core component of the implementation strategy in this study. Although we demonstrated substantial improvements in data quality, national scale-up of this intervention will require significant financial investment for repeated provider training, routine supportive supervision, data management, and system integration. In addition, limited digital infrastructure, electricity, internet connectivity, and the availability of trained digital health personnel in many DHs may pose critical practical challenges for future digitisation and large-scale implementation. Sustained capacity building and routine supportive supervision are critical to maintaining high-quality data in resource-limited, high-caseload settings. Strategic human resource planning, including appointing dedicated data focal persons, will help sustain improvements. Additionally, the gradual adoption of electronic registers tailored to the local context could further strengthen data management and support evidence-based maternal health policies.

Adaptation of this intervention to primary- and tertiary-level health care facilities would require important structural modifications. At the primary-care level, where delivery volumes are lower, and specialist obstetric staff and anaesthesiologists are limited, task-shifting to midwives and nurses, and integration within routine maternal registers would be required. In contrast, tertiary hospitals manage high patient volumes, complex referrals, and multiple parallel documentation systems; therefore, successful adaptation would require stronger supervision mechanisms, dedicated data personnel, and integration with electronic hospital information systems where available. Tailored training intensity, human resource capacity, and monitoring structures would need to be modified according to the level of care.

### Strengths and limitations

The first key strength of our study is that, to the best of our knowledge, it is the first to describe the implementation of the Robson TGCS at secondary health care facilities in Bangladesh and across Southeast Asia. Second, the comprehensive stakeholder engagement throughout the implementation process ensures a collaborative and effective approach to improving data quality. Third, we selected facilities with both high and low patient burdens to explore the applicability of Robson TGCS across diverse health care settings. Fourthly, the inclusion of eight DHs from different divisions ensures a broad representation of the country’s secondary health care landscape. This distinction highlights the innovative nature of our research and its potential to serve as a model for similar settings.

However, the study was limited to secondary-level health care facilities, which restricts the generalisability of findings to other levels of care. Due to time and resource constraints, we did not investigate the cost and sustainability of implementing Robson TGCS. We recommend that future studies assess the cost-effectiveness of implementing the Robson classification. This research could provide valuable insights into the financial implications, long-term feasibility, and potential to address challenges highlighted in this study, particularly in resource-limited settings. Additionally, our study included only pregnancies of 28 weeks or more, based on the national definition of viability in Bangladesh. As many developed and some developing countries apply the Robson classification from 20 or 22 weeks of gestation, this difference in gestational age inclusion may limit the comparability of our findings with studies from those settings. As the study relied on routine register data, some recording errors may have remained, particularly in facilities with a high patient workload. Although documentation improved during the study period, sustaining these improvements may be challenging if routine supervisory and technical support are reduced after the intervention period.

## CONCLUSIONS

Implementing the Robson TGCS in resource-limited countries like Bangladesh requires a systematic approach to overcome challenges related to reporting, data quality, and consistency. Active participation from local and national stakeholders, supported by technical experts, is essential to maintain continuous data quality. Accurate data are essential for determining group-specific c-section rates and planning targeted interventions. Globally, this approach can serve as a model for countries like Bangladesh facing similar challenges, helping to optimise c-sections through data-driven, group-specific interventions, ultimately improving maternal and newborn health outcomes.

## Additional material


Online Supplementary Document
