# Supplementary material for: Effect of an integrated intervention on the availability and completeness of Robson ten group classification system-related data in district hospitals in Bangladesh
Source: J Glob Health. 2026 Feb 27;16:04069. doi: 10.7189/jogh.16.04069 (PMC12947717; doi:10.7189/jogh.16.04069)
Supplement: Online Supplementary Document [file jogh-16-04069-s001.pdf]

Supplement to: Hossain L, Hasan ASM, Mahmood HR, Azrin F, Ahmed A, Sayeed A, Jabeen S, Akter E, Murshid HB, Hassan AKMM, Rahman MM, Mallick T, Tonmon TT, Siddique MAB, Zaman S, Rasghuvanshi VS, Rahman A, Nadia N, Mahmud M, Alim MA, Rahman AE, Hoque DME, Arifeen SE. Effect of an integrated intervention on the availability and completeness of Robson ten group classification system-related data in district hospitals in Bangladesh. J Glob Health. 2026;16:04069.

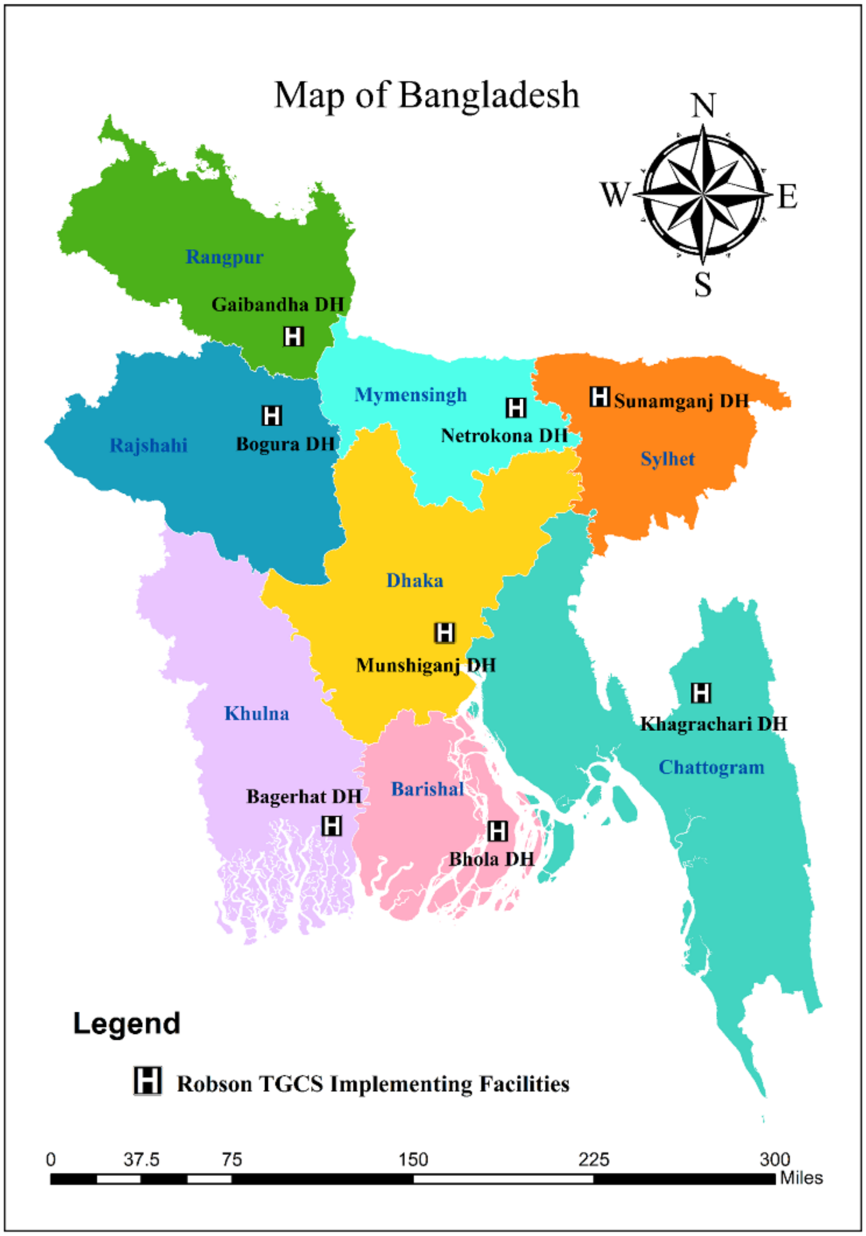

Figure S1: Geographic locations of study facilities across selected district hospitals in Bangladesh

Sl No.	Facility Name	Basic Information of Study Area					Selection Criteria of Study Facility	
		District Name	Division Name	Area (sq. km)	No. of Upazilas	Total Women of Reproductive Age	Total delivery	ObGyn and Anesthesiologist Pair
1	Munshiganj DH	Munshiganj	Dhaka	2898.7	12	1,354,464	785	Yes
2	Bogura DH	Bogura	Rajshahi	2699.6	8	223,928	743	Yes
3	Gaibandha DH	Gaibandha	Rangpur	3959.1	9	587,658	1234	Yes
4	Netrokona DH	Netrokona	Mymenshing	3403.5	7	654,965	2468	Yes
5	Sunamganj DH	Sunamganj	Sylhet	1004.3	6	572,745	2223	Yes
6	Bagerhat DH	Bagerhat	Khulna	3747.2	11	865,222	463	Yes
7	Bhola DH	Bhola	Barishal	2179.3	7	926,811	1426	Yes
8	Khagrachari DH	Khagrachari	Chattogram	2794.3	10	811,171	2081	Yes

Table S1: Basic information of study area and selection criteria of study facility from DHIS2 (1) and Hospital Health Workforce (HWF) (2)

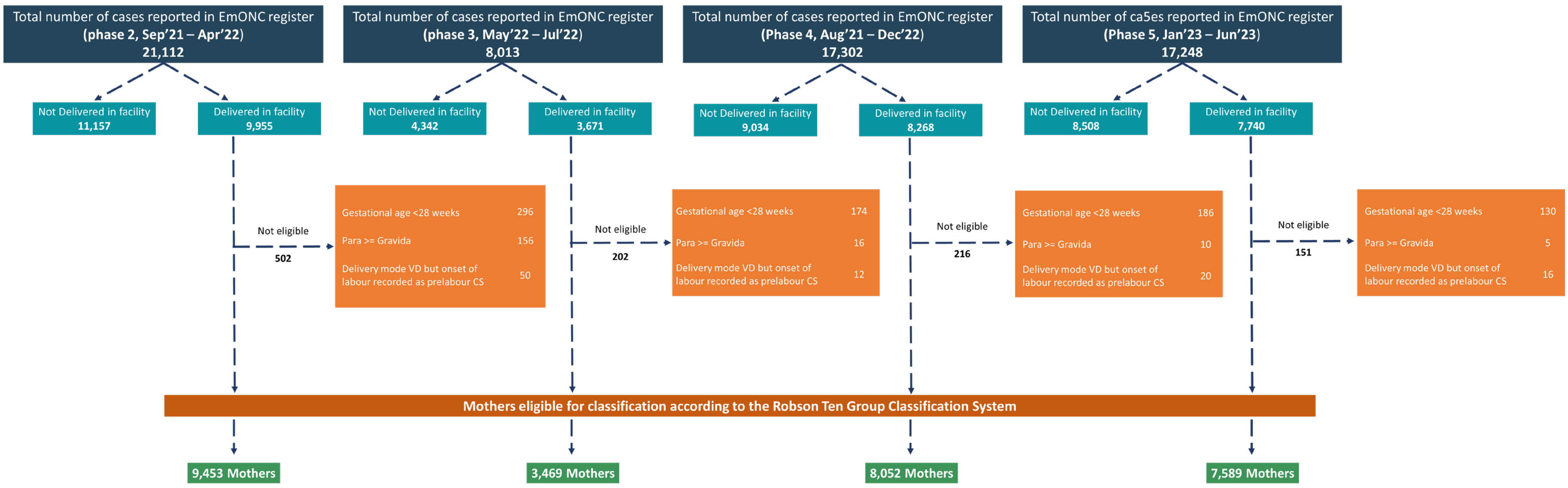

Figure S2: Study flowchart

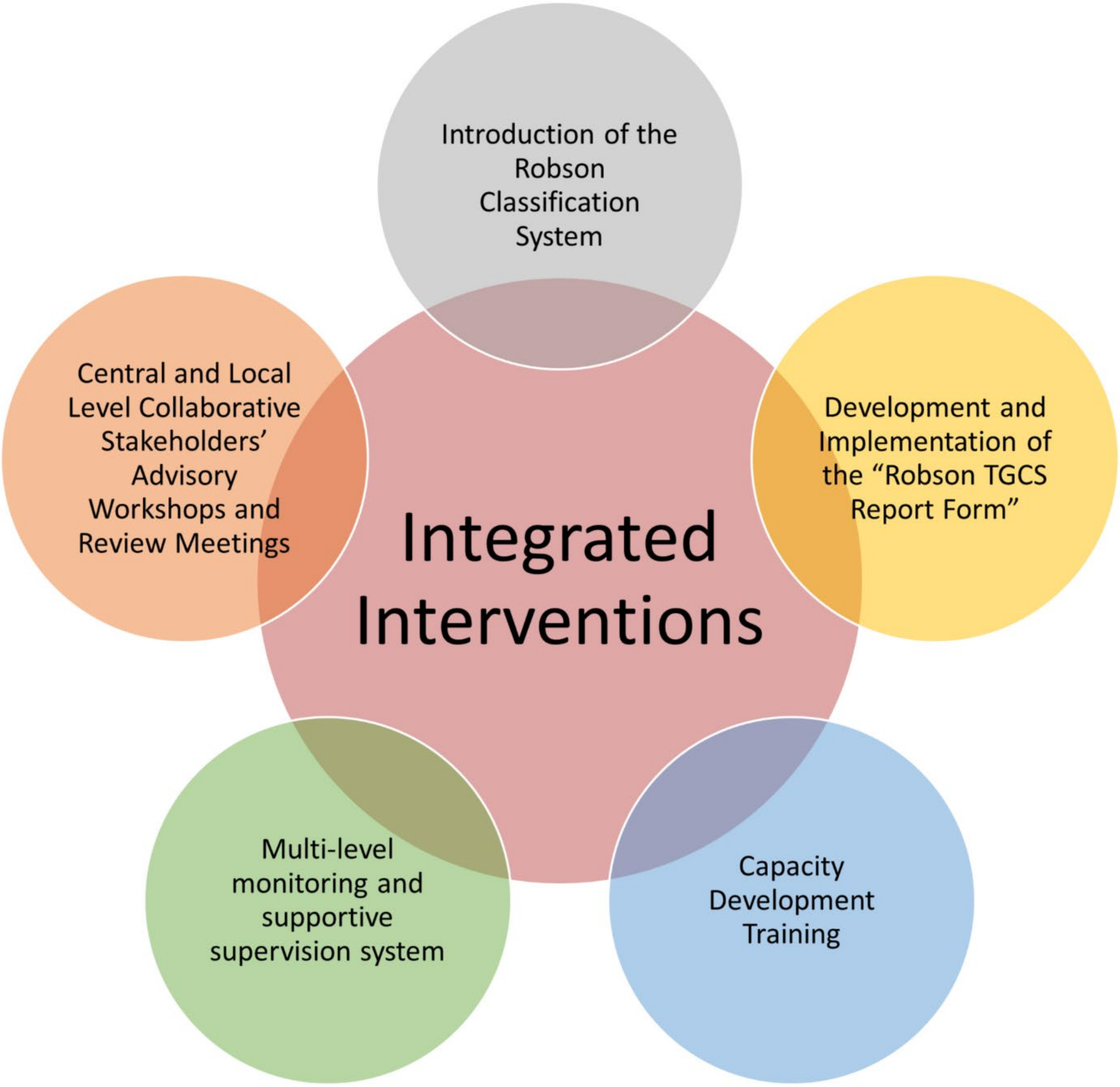

Figure S3: . Key Components of Integrated Interventions to Enhance Availability and Completeness of Robson TGCS-Related Data

	Phase 1: Identification (Jan' 21-Jul'21)							Phase 2: Sensitisation (Aug' 21-Apr'22)									Phase 3: Involvement (May' 22-July'22)			Phase 4: Engagement (Aug' 22- Dec'22)					Phase 5: Engagement (Jan' 23- Jun'23)					
	Jan	Feb	Mar	Apr	May	Jun	Jul	Aug	Sep	Oct	Nov	Dec	Jan	Feb	Mar	Apr	May	Jun	Jul	Aug	Sep	Oct	Nov	Dec	Jan	Feb	Mar	Apr	May	Jun
Study Approval from MNCAH (DGHS)	✓																													
Desk Review	✓	✓	✓																											
Variable Mapping in existing registers for Robson TGCS			✓	✓																										
Stakeholder Identification Workshop (#2)					✓		✓																							
Facility identification Meeting (#1)						✓																								
Central Level Sensitisation meeting (#1)								✓																						
"Robson TGCS Report Form" Review and Finalisation Meeting (#2)								✓																						
Local Level Sensitisation meeting (#1)								✓																						
Local Level "Robson TGCS Report Form" Orientation Meeting in 8DHs (#1)								✓																						
Online Training on Robson TGCS (#3)								✓	✓																					
Weekly Monitoring and supervision visits By DSRHRO									✓	✓	✓	✓	✓	✓	✓	✓	✓	✓	✓	✓	✓	✓	✓	✓	✓	✓	✓	✓	✓	✓
Monthly Data collection started									✓	✓	✓	✓	✓	✓	✓	✓	✓	✓	✓	✓	✓	✓	✓	✓	✓	✓	✓	✓	✓	✓
Findings sharing meeting with central-level stakeholders (#3)																✓			✓					✓						
Local-level Findings sharing meeting (#1)																✓														
F2F training sessions on the "Robson TGCS", "EmONC Register" and "Robson TGCS Report Form" in 8 DHs (#8)																	✓	✓												
Coordination meeting with central-level stakeholders (#1)																	✓													
Daily Data monitoring by ObGyn consultants or designated personnel																	✓	✓	✓	✓	✓	✓	✓	✓	✓	✓	✓	✓	✓	✓
Biweekly visit by icddr,b research team																	✓	✓	✓	✓	✓	✓	✓	✓	✓	✓	✓	✓	✓	✓
Quarterly review meeting (#32) with Facility managers & HCPs																	✓	✓			✓	✓			✓	✓			✓	✓
Refresher F2F training sessions on the "Robson TGCS", "EmONC Register" and "Robson TGCS Report Form" in 8 DHs (#8)																				✓	✓									
Quarterly supportive supervision visits (#24) by OGSB and MNC&AH in 8 DHs																					✓	✓			✓	✓			✓	✓
F2F training sessions on the "Robson TGCS", "EmONC Register" and "Robson TGCS Report Form" in Bhola DH (#1)																										✓				
F2F training sessions on the "Robson TGCS", "EmONC Register" and "Robson TGCS Report Form" in Bogura DH (#1)																											✓			
Central level Findings sharing Workshop (#2)																										✓	✓			

Central Level activities

Local Level activities

Figure S4: . Timeline of phase-specific integrated interventions to enhance Robson TGCS data availability and completeness

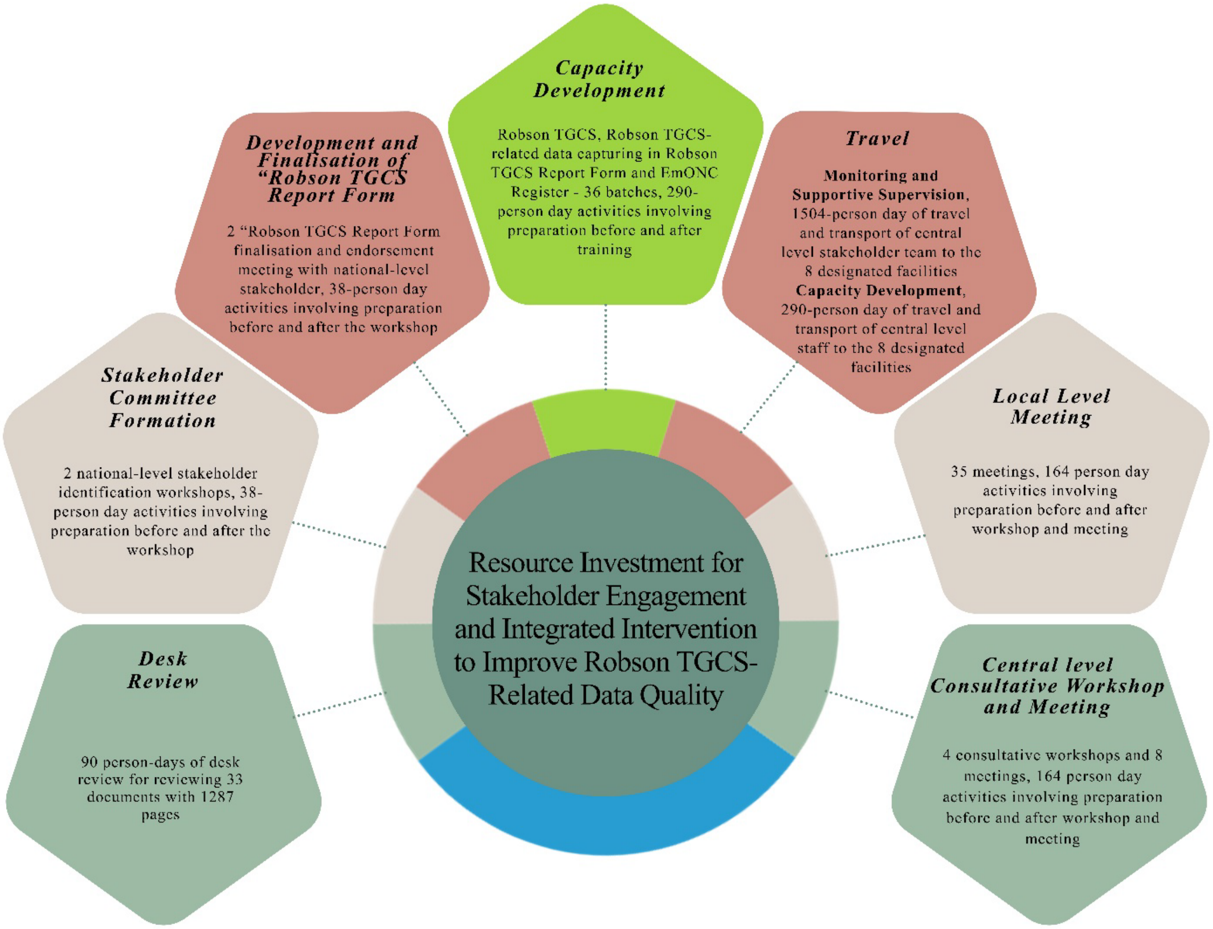

Figure S5: . Resource Investment for Stakeholder Engagement and Integrated Intervention to Improve Robson TGCS-Related Data Quality

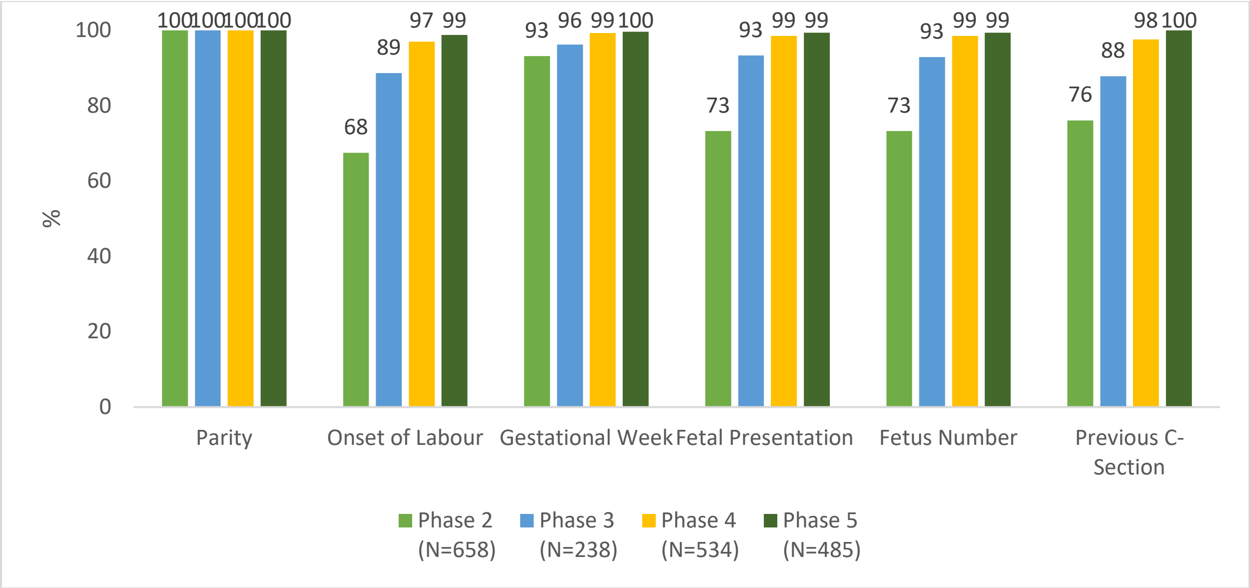

Figure S6: Phase-wise availability (%) of Robson TGCS–related variables in Munshiganj DH

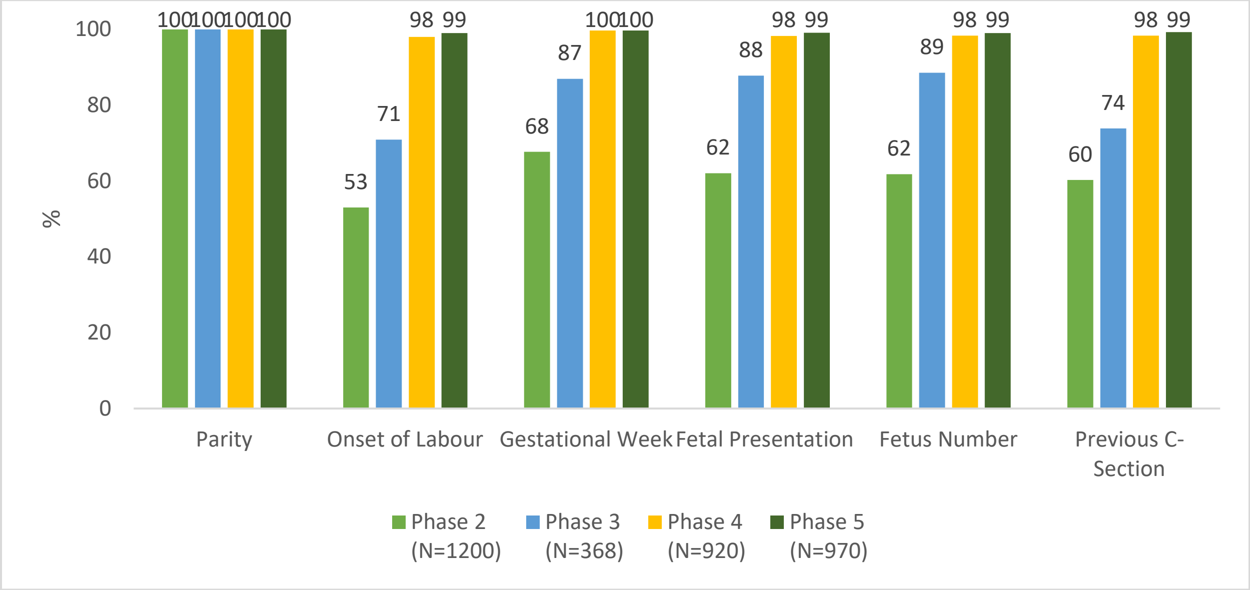

Figure S7: Phase-wise availability (%) of Robson TGCS–related variables in Bogura DH

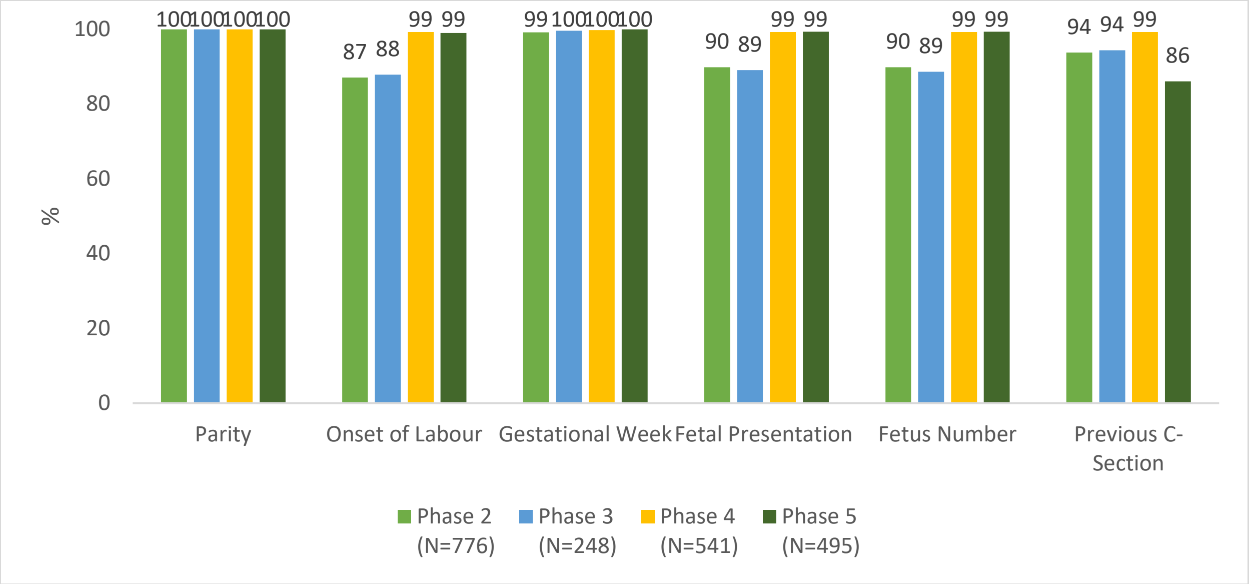

Figure S8: Phase-wise availability (%) of Robson TGCS–related variables in Gaibandha DH

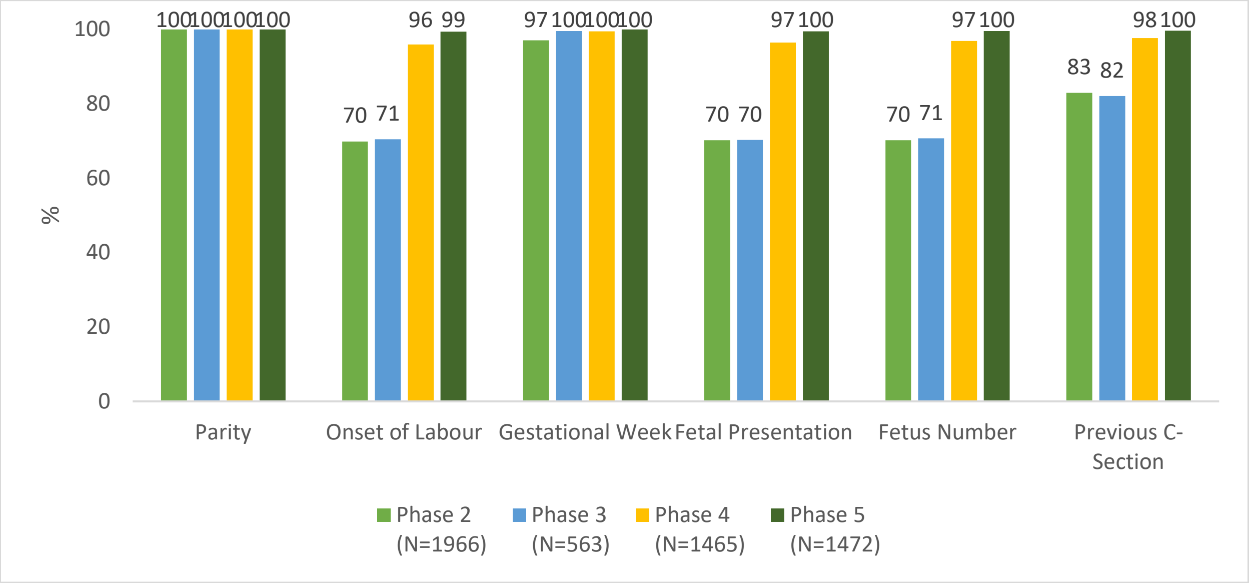

Figure S9: Phase-wise availability (%) of Robson TGCS–related variables in Netrokona DH

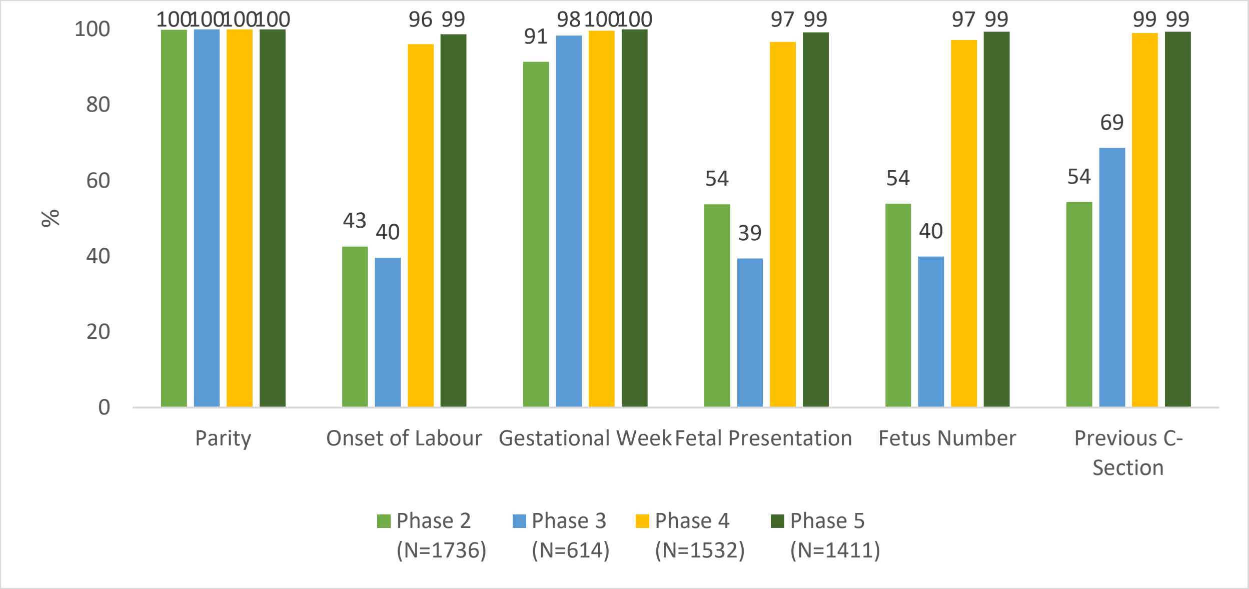

Figure S10: Phase-wise availability (%) of Robson TGCS–related variables in Sunamganj DH

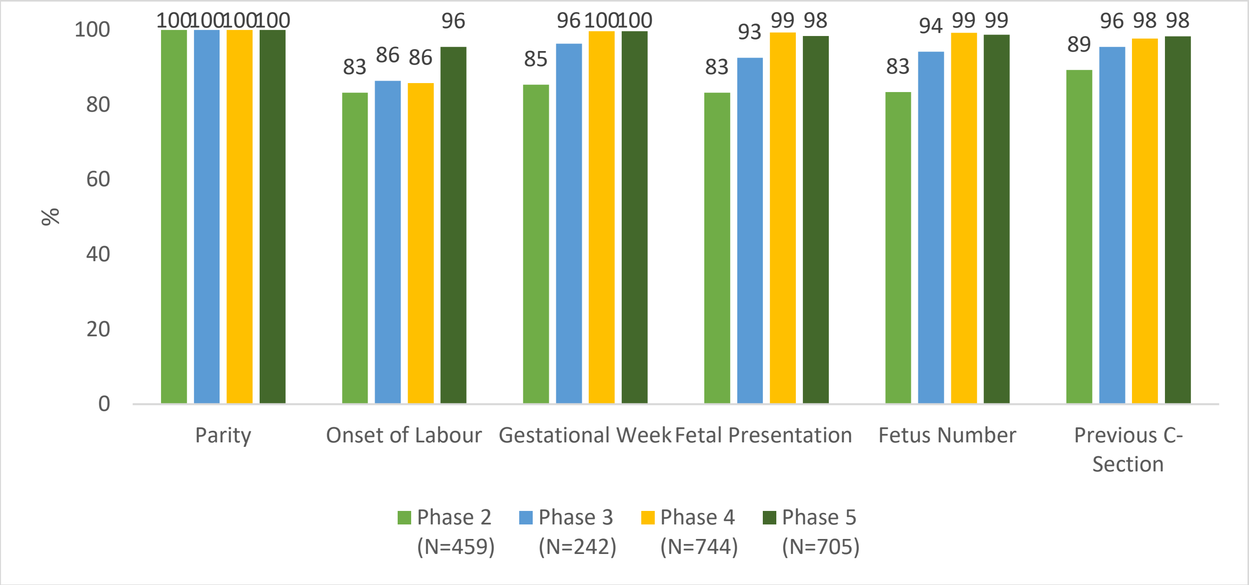

Figure S11: Phase-wise availability (%) of Robson TGCS–related variables in Bagerhat DH

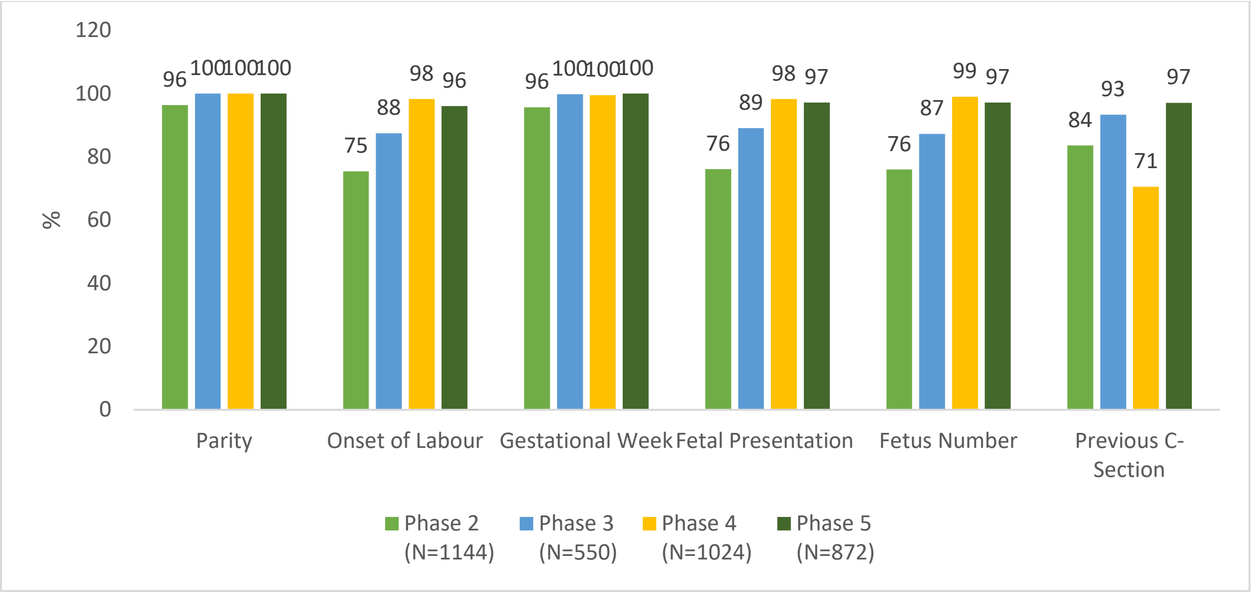

Figure S12: Phase-wise availability (%) of Robson TGCS–related variables in Bhola DH

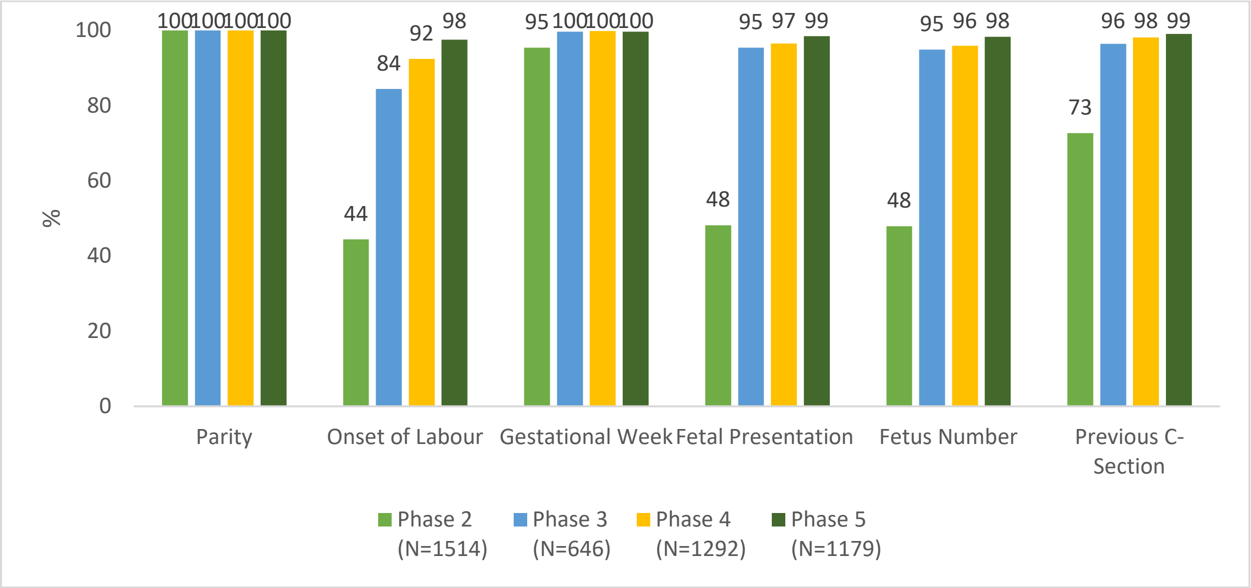

Figure S13: Phase-wise availability (%) of Robson TGCS–related variables in Khagrachari DH

Variables	Phase 2			Phase 3			Phase 4			Phase 5		
	Percentage	LCI	UCI	Percentage	LCI	UCI	Percentage	LCI	UCI	Percentage	LCI	UCI
Parity	100	99.4	99.7	100	99.9	100.0	100	100.0	100.0	100	100.0	100.0
Onset of Labour	61	60.2	62.2	74	72.4	75.4	95	94.8	95.8	98	97.8	98.4
Gestational Week	91	90.6	91.8	98	97.1	98.1	100	99.4	99.7	100	99.7	99.9
Fetal Present	66	64.9	66.8	79	77.4	80.2	98	97.2	97.9	99	98.6	99.1
Fetus No	66	64.8	66.7	79	77.3	80.1	98	97.4	98.0	99	98.6	99.1
Previous CS	74	73.1	74.9	86	84.3	86.6	95	94.2	95.2	98	97.9	98.5
Average variable availability	76	75.7	76.8	86	85.0	86.6	97	97.3	97.7	99	98.8	99.1

Table S2. Availability (%) of Robson TGCS variables with 95% confidence intervals in eight district hospitals.

Variables	Phase 2			Phase 3			Phase 4			Phase 5		
	Percentage	LCI	UCI	Percentage	LCI	UCI	Percentage	LCI	UCI	Percentage	LCI	UCI
Parity	100	99.4	100.0	100	98.5	100.0	100	99.3	100.0	100	99.2	100.0
Onset of Labour	68	63.7	71.0	89	83.9	92.4	97	95.2	98.3	99	97.3	99.5
Gestational Week	93	91.0	95.0	96	92.9	98.3	99	98.1	99.8	100	98.5	100.0
Fetal Present	73	69.7	76.6	93	89.3	96.1	99	97.1	99.4	99	98.2	99.9
Fetus No	73	69.7	76.6	93	88.8	95.8	99	97.1	99.4	99	98.2	99.9
Previous CS	76	72.7	79.3	88	83.0	91.7	98	95.9	98.7	100	99.2	100.0
Average variable availability	81	78.5	82.6	93	91.0	95.3	98	97.8	99.2	100	99.1	100.0

Table S3: Availability (%) of Robson TGCS variables with 95% confidence intervals in Munshiganj DH

Variables	Phase 2			Phase 3			Phase 4			Phase 5		
	Percentage	LCI	UCI	Percentage	LCI	UCI	Percentage	LCI	UCI	Percentage	LCI	UCI
Parity	100	99.7	100.0	100	99.0	100.0	100	99.6	100.0	100	99.6	100.0
Onset of Labour	53	50.1	55.9	71	66.0	75.5	98	96.9	98.8	99	98.1	99.5
Gestational Week	68	65.0	70.4	87	83.1	90.2	100	99.1	99.9	100	99.1	99.9
Fetal Present	62	59.3	64.8	88	84.0	90.9	98	97.2	99.0	99	98.2	99.6
Fetus No	62	59.0	64.6	89	84.9	91.6	98	97.3	99.1	99	98.1	99.5
Previous CS	60	57.5	63.1	74	69.1	78.3	98	97.3	99.1	99	98.5	99.7
Average variable availability	68	65.9	69.1	85	82.7	86.6	99	98.3	99.3	99	98.9	99.7

Table S4: Availability (%) of Robson TGCS variables with 95% confidence intervals in Bogura DH

Variables	Phase 2			Phase 3			Phase 4			Phase 5		
	Percentage	LCI	UCI	Percentage	LCI	UCI	Percentage	LCI	UCI	Percentage	LCI	UCI
Parity	100	99.5	100.0	100	98.5	100.0	100	99.3	100.0	100	99.3	100.0
Onset of Labour	87	84.6	89.4	88	83.2	91.7	99	98.1	99.8	99	97.7	99.7
Gestational Week	99	98.3	99.7	100	97.8	100.0	100	99.0	100.0	100	99.3	100.0
Fetal Present	90	87.6	92.0	89	84.6	92.7	99	98.1	99.8	99	98.2	99.9
Fetus No	90	87.6	92.0	89	84.1	92.4	99	98.1	99.8	99	98.2	99.9
Previous CS	94	91.9	95.4	94	90.7	96.9	99	98.1	99.8	86	82.7	89.0
Average variable availability	93	92.0	94.6	93	90.9	95.6	99	99.0	100.0	97	96.7	97.9

Table S5: Availability (%) of Robson TGCS variables with 95% confidence intervals in Gaibandha DH

Variables	Phase 2			Phase 3			Phase 4			Phase 5		
	Percentage	LCI	UCI	Percentage	LCI	UCI	Percentage	LCI	UCI	Percentage	LCI	UCI
Parity	100	99.8	100.0	100	99.3	100.0	100	99.7	100.0	100	99.7	100.0
Onset of Labour	70	67.8	71.9	71	66.6	74.3	96	94.9	97.0	99	98.8	99.7
Gestational Week	97	96.3	97.8	100	98.7	100.0	100	98.9	99.8	100	99.7	100.0
Fetal Present	70	68.2	72.3	70	66.4	74.1	97	95.4	97.4	100	98.9	99.8
Fetus No	70	68.2	72.3	71	66.7	74.4	97	95.8	97.7	100	99.1	99.9
Previous CS	83	81.2	84.6	82	78.6	85.1	98	96.8	98.4	100	99.3	99.9
Average variable availability	82	80.5	83.0	82	79.9	84.5	98	97.2	98.3	100	99.5	100.0

Table S6: Availability (%) of Robson TGCS variables with 95% confidence intervals in Netrokona DH

Variables	Phase 2			Phase 3			Phase 4			Phase 5		
	Percentage	LCI	UCI	Percentage	LCI	UCI	Percentage	LCI	UCI	Percentage	LCI	UCI
Parity	100	99.7	100.0	100	99.4	100.0	100	99.8	100.0	100	99.7	100.0
Onset of Labour	43	40.2	44.9	40	35.7	43.6	96	95.0	97.0	99	97.9	99.2
Gestational Week	91	90.0	92.7	98	97.0	99.2	100	99.1	99.9	100	99.7	100.0
Fetal Present	54	51.4	56.1	39	35.5	43.4	97	95.7	97.6	99	98.6	99.6
Fetus No	54	51.5	56.2	40	36.0	43.9	97	96.2	98.0	99	98.8	99.7
Previous CS	54	51.9	56.7	69	64.7	72.2	99	98.4	99.5	99	98.9	99.8
Average variable availability	66	64.7	67.3	64	62.0	66.6	98	97.6	98.6	99	99	99.7

Table S7: Availability (%) of Robson TGCS variables with 95% confidence intervals in Sunamganj DH

Variables	Phase 2			Phase 3			Phase 4			Phase 5		
	Percentage	LCI	UCI	Percentage	LCI	UCI	Percentage	LCI	UCI	Percentage	LCI	UCI
Parity	100	99.2	100.0	100	98.5	100.0	100	99.5	100.0	100	99.5	100.0
Onset of Labour	83	79.5	86.5	86	81.4	90.4	86	83.0	88.2	96	93.7	96.9
Gestational Week	85	81.8	88.5	96	93.1	98.3	100	99.0	100.0	100	99.0	100.0
Fetal Present	83	79.5	86.5	93	88.5	95.5	99	98.4	99.8	98	97.2	99.2
Fetus No	83	79.7	86.7	94	90.5	96.8	99	98.3	99.7	99	97.6	99.4
Previous CS	89	86.1	92.0	96	92.0	97.7	98	96.4	98.7	98	97.0	99.1
Average variable availability	87	85.0	89.9	94	92.1	96.2	97	96.4	97.5	98	97.9	99.0

Table S8: Availability (%) of Robson TGCS variables with 95% confidence intervals in Bagerhat DH

Variables	Phase 2			Phase 3			Phase 4			Phase 5		
	Percentage	LCI	UCI	Percentage	LCI	UCI	Percentage	LCI	UCI	Percentage	LCI	UCI
Parity	96	95.2	97.4	100	99.3	100.0	100	99.6	100.0	100	99.6	100.0
Onset of Labour	75	72.8	77.9	88	84.4	90.1	98	97.4	99.0	96	94.6	97.3
Gestational Week	96	94.4	96.8	100	99.0	100.0	100	98.9	99.8	100	99.6	100.0
Fetal Present	76	73.6	78.6	89	86.2	91.6	98	97.4	99.0	97	95.9	98.2
Fetus No	76	73.4	78.4	87	84.2	89.9	99	98.2	99.5	97	95.9	98.2
Previous CS	84	81.3	85.7	93	90.8	95.2	71	67.6	73.3	97	95.8	98.1
Average variable availability	84	82.2	85.6	93	91.2	94.4	94	93.7	94.9	98	97.3	98.6

Table S9: Availability (%) of Robson TGCS variables with 95% confidence intervals in Bhola DH

Variables	Phase 2			Phase 3			Phase 4			Phase 5		
	Percentage	LCI	UCI	Percentage	LCI	UCI	Percentage	LCI	UCI	Percentage	LCI	UCI
Parity	100	99.8	100.0	100	99.4	100.0	100	99.7	100.0	100	99.7	100.0
Onset of Labour	44	41.9	46.9	84	81.3	87.1	92	90.8	93.8	98	96.5	98.3
Gestational Week	95	94.2	96.4	100	98.9	100.0	100	99.3	100.0	100	99.1	99.9
Fetal Present	48	45.7	50.8	95	93.4	96.8	97	95.4	97.4	99	97.6	99.1
Fetus No	48	45.3	50.4	95	92.9	96.5	96	94.7	96.9	98	97.4	99.0
Previous CS	73	70.3	74.9	96	94.7	97.7	98	97.2	98.7	99	98.3	99.5
Average variable availability	68	66.6	69.6	95	94.1	96.1	97	96.5	97.7	99	98.4	99.3

Table S10: Availability (%) of Robson TGCS variables with 95% confidence intervals in Khagrachari DH

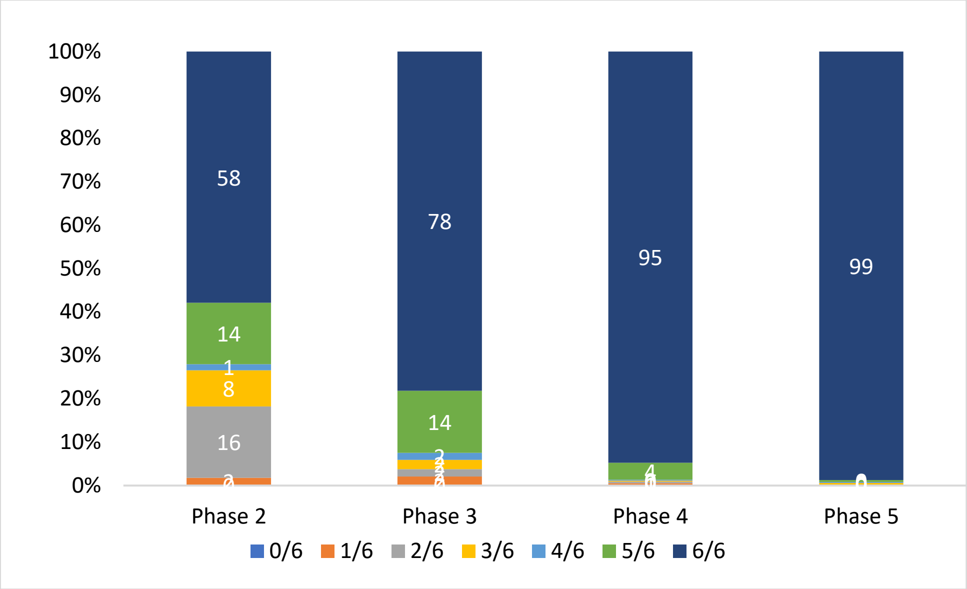

Figure S14: Phase-wise completeness (%) of six Robson TGCS–related variables in Munshiganj DH

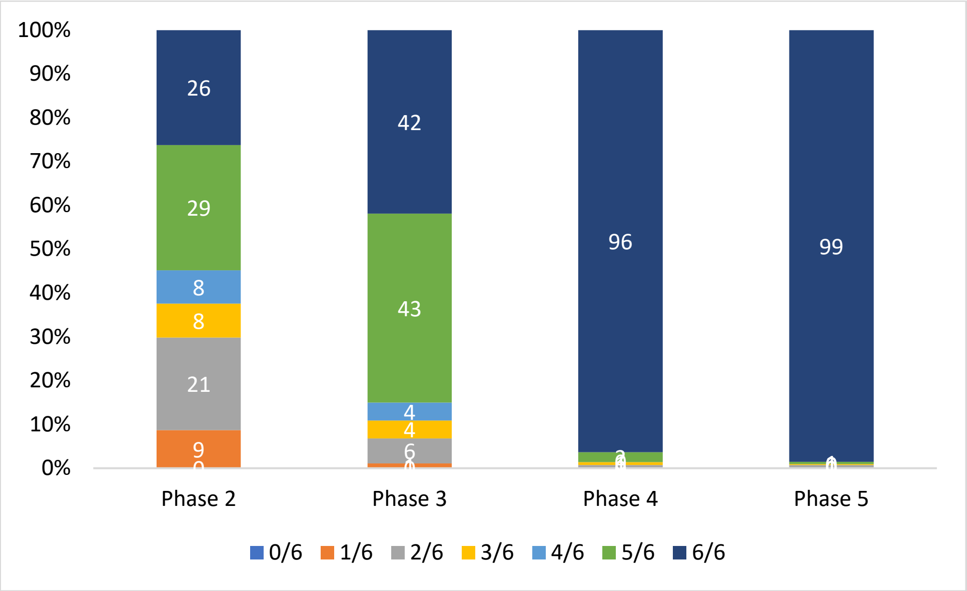

Figure S15: Phase-wise completeness (%) of six Robson TGCS–related variables in Bogura DH

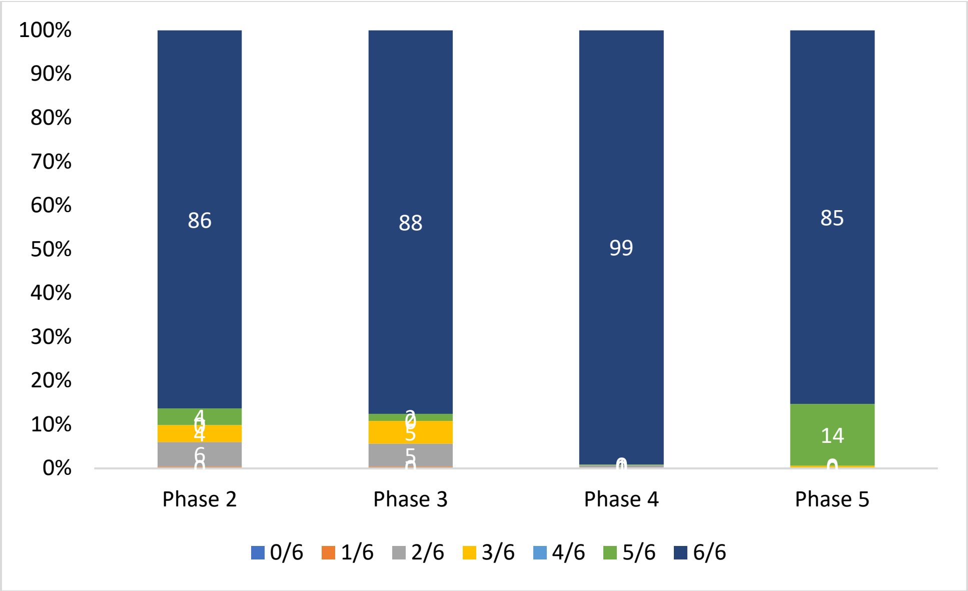

Figure S16: Phase-wise completeness (%) of six Robson TGCS–related variables in Gaibandha DH

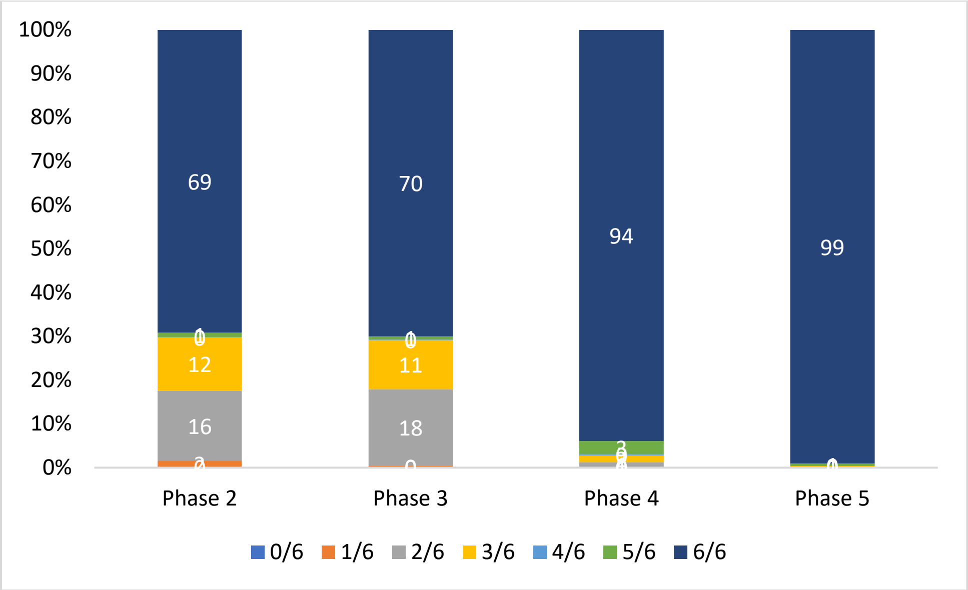

Figure S17: Phase-wise completeness (%) of six Robson TGCS–related variables in Netrokona DH

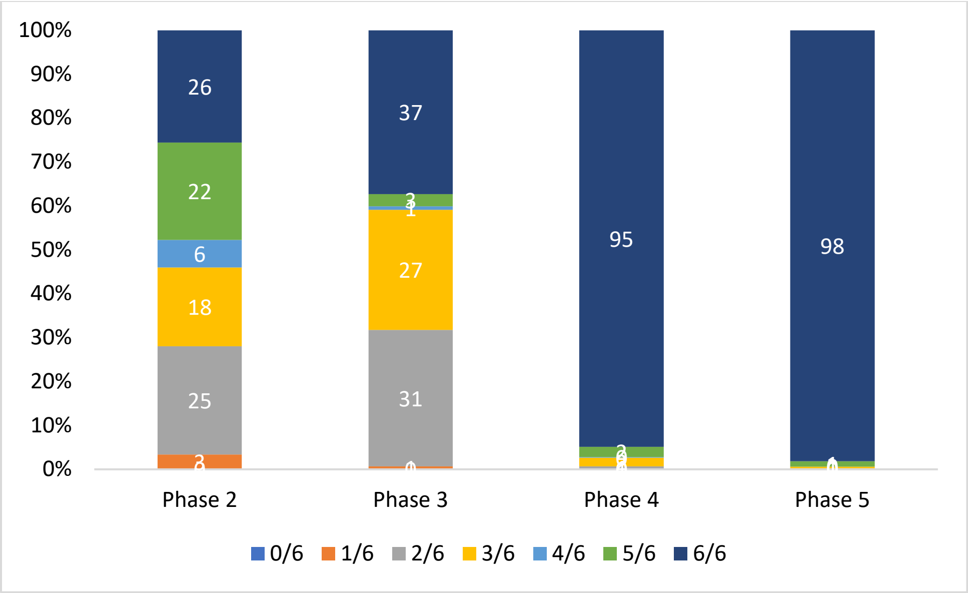

Figure S18: Phase-wise completeness (%) of six Robson TGCS–related variables in Sunamganj DH

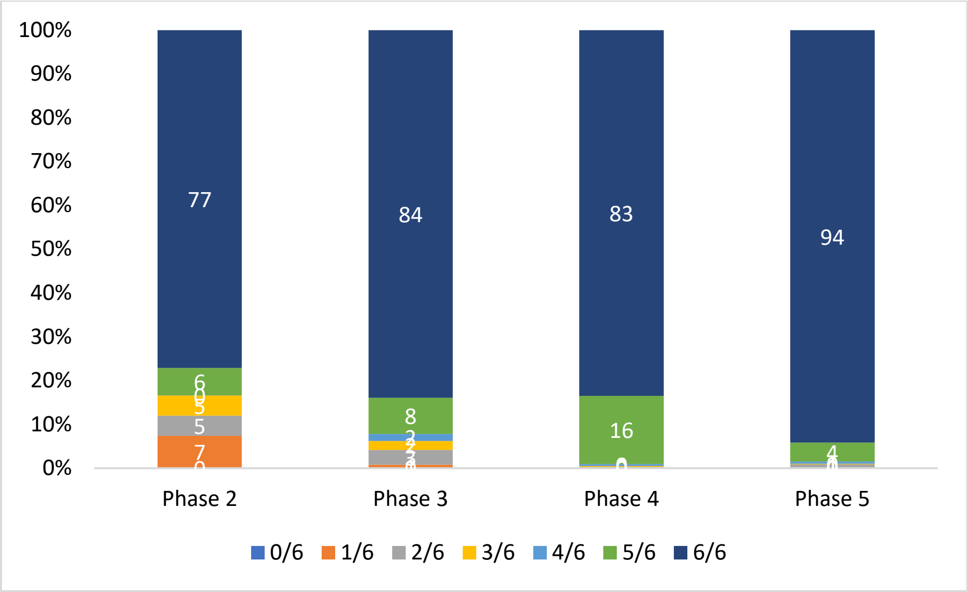

Figure S19: Phase-wise completeness (%) of six Robson TGCS–related variables in Bagerhat DH

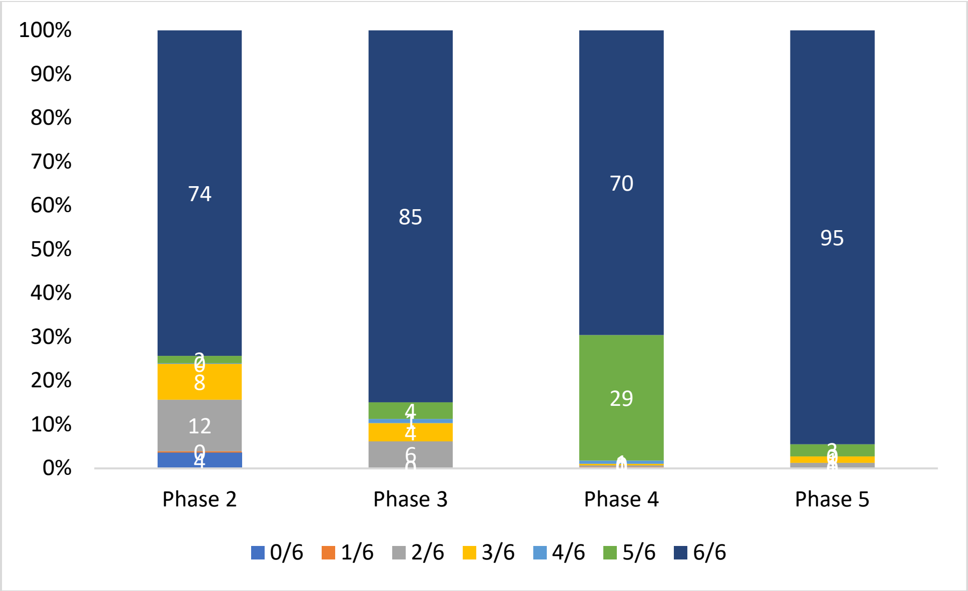

Figure S20: Phase-wise completeness (%) of six Robson TGCS–related variables in Bhola DH

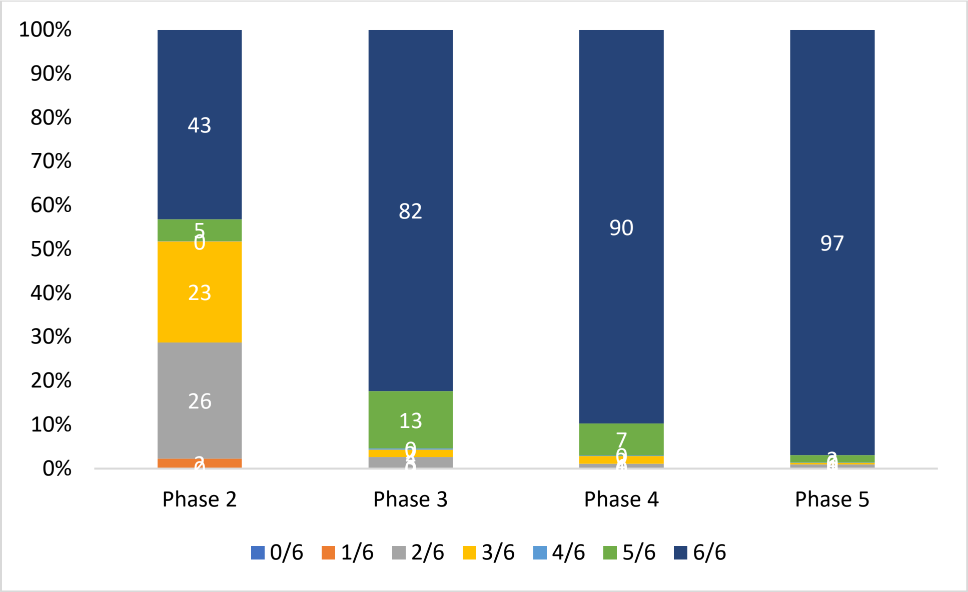

Figure S21: Phase-wise completeness (%) of six Robson TGCS–related variables in Khagrachari DH

Number of variable	Phase 2			Phase 3			Phase 4			Phase 5		
	Percentage	LCL	UCL	Percentage	LCL	UCL	Percentage	LCL	UCL	Percentage	LCL	UCL
0	0.4	0.3	0.6	0.0	0.0	0.0	0.0	0.0	0.0	0.0	0.0	0.0
1	3.0	2.7	3.3	0.5	0.3	0.9	0.1	0.1	0.2	0.0	0.0	0.1
2	18.0	17.3	18.8	11.1	10.1	12.2	0.7	0.5	0.9	0.5	0.4	0.7
3	12.6	12.0	13.3	8.7	7.8	9.7	1.1	0.9	1.4	0.5	0.3	0.7
4	2.2	2.0	2.6	1.0	0.7	1.4	0.2	0.2	0.4	0.1	0.0	0.2
5	10.5	9.9	11.2	9.9	8.9	10.9	7.8	7.2	8.4	2.3	2.0	2.7
6	53.2	52.2	54.2	68.7	67.1	70.2	90.0	89.4	90.7	96.6	96.2	97.0

Table S11. Completeness (%) of Robson TGCS variables with 95% confidence intervals by phase in eight district hospitals, Bangladesh.

Number of variable	Phase 2			Phase 3			Phase 4			Phase 5		
	Percentage	LCL	UCL	Percentage	LCL	UCL	Percentage	LCL	UCL	Percentage	LCL	UCL
0	0.0	0.0	0.0	0.0	0.0	0.0	0.0	0.0	0.0	0.0	0.0	0.0
1	1.8	1.0	3.2	2.1	0.9	5.0	0.6	0.2	1.7	0.0	0.0	0.0
2	16.4	13.8	19.5	1.7	0.6	4.4	0.4	0.1	1.5	0.2	0.0	1.5
3	8.4	6.5	10.7	2.1	0.9	5.0	0.2	0.0	1.3	0.4	0.1	1.6
4	1.4	0.7	2.6	1.7	0.6	4.4	0.2	0.0	1.3	0.2	0.0	1.5
5	14.1	11.7	17.0	14.3	10.4	19.4	3.9	2.6	6.0	0.4	0.1	1.6
6	57.9	54.1	61.6	78.2	72.4	83.0	94.8	92.5	96.4	98.8	97.3	99.4

Table S12: Completeness (%) of Robson TGCS variables with 95% confidence intervals by phase in Munshiganj DH

Number of variable	Phase 2			Phase 3			Phase 4			Phase 5		
	Percentage	LCL	UCL	Percentage	LCL	UCL	Percentage	LCL	UCL	Percentage	LCL	UCL
0	0.0	0.0	0.0	0.0	0.0	0.0	0.0	0.0	0.0	0.0	0.0	0.0
1	8.7	7.2	10.4	1.1	0.4	2.9	0.0	0.0	0.0	0.0	0.0	0.0
2	21.2	18.9	23.6	5.7	3.7	8.6	0.8	0.4	1.6	0.7	0.3	1.5
3	7.8	6.4	9.4	4.1	2.5	6.7	0.7	0.3	1.4	0.2	0.1	0.8
4	7.6	6.2	9.2	4.1	2.5	6.7	0.0	0.0	0.0	0.0	0.0	0.0
5	28.6	26.1	31.2	43.2	38.2	48.3	2.3	1.5	3.5	0.5	0.2	1.2
6	26.3	23.8	28.8	41.8	36.9	47.0	96.3	94.9	97.3	98.6	97.6	99.1

Table S13: Completeness (%) of Robson TGCS variables with 95% confidence intervals by phase in Bogura DH

Number of variable	Phase 2			Phase 3			Phase 4			Phase 5		
	Percentage	LCL	UCL	Percentage	LCL	UCL	Percentage	LCL	UCL	Percentage	LCL	UCL
0	0.0	0.0	0.0	0.0	0.0	0.0	0.0	0.0	0.0	0.0	0.0	0.0
1	0.4	0.1	1.2	0.4	0.1	2.8	0.0	0.0	0.0	0.0	0.0	0.0
2	5.7	4.2	7.5	5.2	3.1	8.8	0.7	0.3	2.0	0.2	0.0	1.4
3	3.9	2.7	5.5	5.2	3.1	8.8	0.0	0.0	0.0	0.4	0.1	1.6
4	0.0	0.0	0.0	0.0	0.0	0.0	0.0	0.0	0.0	0.0	0.0	0.0
5	3.7	2.6	5.3	1.6	0.6	4.2	0.2	0.0	1.3	14.1	11.3	17.5
6	86.3	83.7	88.6	87.5	82.7	91.1	99.1	97.8	99.6	85.3	81.8	88.1

Table S14: Completeness (%) of Robson TGCS variables with 95% confidence intervals by phase in Gaibandha DH

Number of variable	Phase 2			Phase 3			Phase 4			Phase 5		
	Percentage	LCL	UCL	Percentage	LCL	UCL	Percentage	LCL	UCL	Percentage	LCL	UCL
0	0.0	0.0	0.0	0.0	0.0	0.0	0.0	0.0	0.0	0.0	0.0	0.0
1	1.6	1.2	2.3	0.4	0.1	1.4	0.2	0.1	0.6	0.1	0.0	0.5
2	15.9	14.4	17.6	17.6	14.6	21.0	1.1	0.7	1.8	0.3	0.1	0.8
3	12.2	10.8	13.7	11.2	8.8	14.1	1.5	1.0	2.3	0.0	0.0	0.0
4	0.0	0.0	0.0	0.2	0.0	1.3	0.3	0.1	0.7	0.0	0.0	0.0
5	1.1	0.7	1.7	0.7	0.3	1.9	3.0	2.2	4.0	0.5	0.3	1.1
6	69.1	67.0	71.1	70.0	66.1	73.6	93.9	92.6	95.0	99.0	98.4	99.4

Table S15: Completeness (%) of Robson TGCS variables with 95% confidence intervals by phase in Netrokona DH

Number of variable	Phase 2			Phase 3			Phase 4			Phase 5		
	Percentage	LCL	UCL	Percentage	LCL	UCL	Percentage	LCL	UCL	Percentage	LCL	UCL
0	0.0	0.0	0.0	0.0	0.0	0.0	0.0	0.0	0.0	0.0	0.0	0.0
1	1.6	1.2	2.3	0.4	0.1	1.4	0.2	0.1	0.6	0.1	0.0	0.5
2	15.9	14.4	17.6	17.6	14.6	21.0	1.1	0.7	1.8	0.3	0.1	0.8
3	12.2	10.8	13.7	11.2	8.8	14.1	1.5	1.0	2.3	0.0	0.0	0.0
4	0.0	0.0	0.0	0.2	0.0	1.3	0.3	0.1	0.7	0.0	0.0	0.0
5	1.1	0.7	1.7	0.7	0.3	1.9	3.0	2.2	4.0	0.5	0.3	1.1
6	25.6	23.6	27.7	37.3	33.5	41.2	94.8	93.6	95.8	98.2	97.3	98.7

Table S16: Completeness (%) of Robson TGCS variables with 95% confidence intervals by phase in Sunamganj DH

Number of variable	Phase 2			Phase 3			Phase 4			Phase 5		
	Percentage	LCL	UCL	Percentage	LCL	UCL	Percentage	LCL	UCL	Percentage	LCL	UCL
0	0.0	0.0	0.0	0.0	0.0	0.0	0.0	0.0	0.0	0.0	0.0	0.0
1	7.4	5.3	10.2	0.8	0.2	3.3	0.0	0.0	0.0	0.1	0.0	1.0
2	4.6	3.0	6.9	3.3	1.7	6.5	0.3	0.1	1.1	0.7	0.3	1.7
3	4.6	3.0	6.9	2.1	0.9	4.9	0.3	0.1	1.1	0.1	0.0	1.0
4	0.0	0.0	0.0	1.7	0.6	4.3	0.4	0.1	1.2	0.6	0.2	1.5
5	6.3	4.4	9.0	8.3	5.4	12.5	15.6	13.2	18.4	4.3	3.0	6.0
6	77.1	73.0	80.8	83.9	78.7	88.0	83.5	80.6	86.0	94.2	92.2	95.7

Table S17: Completeness (%) of Robson TGCS variables with 95% confidence intervals by phase in Bagerhat DH

Number of variable	Phase 2			Phase 3			Phase 4			Phase 5		
	Percentage	LCL	UCL	Percentage	LCL	UCL	Percentage	LCL	UCL	Percentage	LCL	UCL
0	3.6	2.6	4.8	0.0	0.0	0.0	0.0	0.0	0.0	0.0	0.0	0.0
1	0.3	0.1	0.9	0.2	0.0	1.3	0.3	0.1	0.9	0.0	0.0	0.0
2	11.7	10.0	13.7	6.0	4.3	8.3	0.4	0.1	1.0	1.3	0.7	2.3
3	8.2	6.8	10.0	4.2	2.8	6.2	0.4	0.1	1.0	1.5	0.9	2.6
4	0.2	0.0	0.7	0.9	0.4	2.2	0.7	0.3	1.4	0.0	0.0	0.0
5	1.7	1.1	2.6	3.8	2.5	5.8	28.7	26.0	31.6	2.8	1.8	4.1
6	74.3	71.7	76.8	84.9	81.7	87.7	69.5	66.6	72.3	94.5	92.8	95.8

Table S18: Completeness (%) of Robson TGCS variables with 95% confidence intervals by phase in Bhola DH

Number of variable	Phase 2			Phase 3			Phase 4			Phase 5		
	Percentage	LCL	UCL	Percentage	LCL	UCL	Percentage	LCL	UCL	Percentage	LCL	UCL
0	0.0	0.0	0.0	0.0	0.0	0.0	0.0	0.0	0.0	0.0	0.0	0.0
1	2.2	1.6	3.1	0.0	0.0	0.0	0.1	0.0	0.5	0.2	0.0	0.7
2	26.5	24.3	28.8	2.6	1.6	4.2	1.0	0.6	1.7	0.8	0.4	1.5
3	23.1	21.0	25.2	1.7	0.9	3.1	1.8	1.2	2.7	0.4	0.2	1.0
4	0.1	0.0	0.5	0.3	0.1	1.2	0.2	0.0	0.6	0.1	0.0	0.6
5	5.0	4.0	6.2	13.0	10.6	15.8	7.3	6.0	8.8	1.6	1.0	2.5
6	43.1	40.7	45.6	82.4	79.2	85.1	89.7	87.9	91.3	96.9	95.8	97.8

Table S19: Completeness (%) of Robson TGCS variables with 95% confidence intervals by phase in Khagrachari DH

Variables	Odds Ratio (OR)	p-value	95% CI	
			Lower	Upper
**Phase**				
Phase 2 (ref)				
Phase 3	2.04	<0.01	1.86	2.24
Phase 4	8.87	<0.01	8.10	9.71
Phase 5	29.35	<0.01	25.55	33.72
**Delivery mode**				
VD (ref)				
CS	0.72	<0.01	0.65	0.78
**Facility name**				
Munshiganj DH(ref)				
Bogura DH	0.40	<0.01	0.34	0.48
Gaibandha DH	2.20	<0.01	1.80	2.69
Netrokona DH	1.25	0.01	1.06	1.46
Sunamganj DH	0.34	<0.01	0.29	0.40
Bagerhat DH	1.05	0.63	0.86	1.28
Bhola DH	0.79	0.01	0.67	0.93
Khagrachari DH	0.51	<0.01	0.44	0.60
**Type of delivery**				
Live birth (ref)				
IUD	0.66	<0.01	0.56	0.77
Still birth	0.44	0.04	0.21	0.96

Table S20: Odds Ratios (ORs) and 95% Confidence Intervals for factors associated with data completeness of Robson TGCS–related variables in eight district hospitals, Bangladesh.

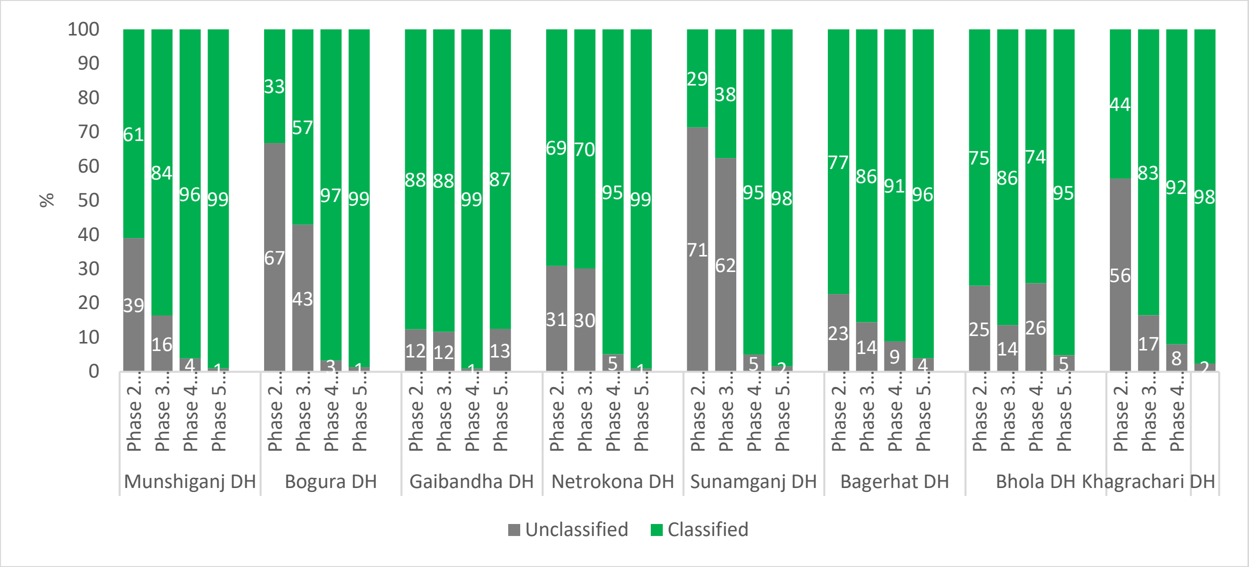

Figure S22: Facility-wise distribution (%) of classified and unclassified Robson groups by phase in eight district hospitals, Bangladesh.

Time Frame	Unclassified			Classified		
	Percentage	LCL	UCL	Percentage	LCL	UCL
Phase 2	45	43.9	45.9	55	54.1	56.1
Phase 3	29	27.2	30.2	71	69.8	72.8
Phase 4	8	7.4	8.6	92	91.4	92.6
Phase 5	3	2.5	3.2	97	96.8	97.5

Table S21. Phase-wise distribution (%) of classified and unclassified Robson groups with 95% confidence intervals in eight district hospitals.

Time Frame	Unclassified			Classified		
	Percentage	LCL	UCL	Percentage	LCL	UCL
Phase 2	39	35.3	42.9	61	57.1	64.7
Phase 3	16	11.9	21.7	84	78.3	88.1
Phase 4	4	2.5	5.9	96	94.1	97.5
Phase 5	1	0.3	2.4	99	97.6	99.7

Table S22: Phase-wise distribution (%) of classified and unclassified Robson groups with 95% confidence intervals in Munshiganj DH

Time Frame	Unclassified			Classified		
	Percentage	LCL	UCL	Percentage	LCL	UCL
**Phase 2**	67	64.1	69.5	33	30.5	35.9
**Phase 3**	43	37.8	48.2	57	51.8	62.2
**Phase 4**	3	2.2	4.6	97	95.4	97.8
**Phase 5**	1	0.7	2.3	99	97.7	99.3

Table S23: Phase-wise distribution (%) of classified and unclassified Robson groups with 95% confidence intervals in Bogura DH

Time Frame	Unclassified			Classified		
	Percentage	LCL	UCL	Percentage	LCL	UCL
**Phase 2**	12	10.1	14.9	88	85.1	89.9
**Phase 3**	12	8.0	16.4	88	83.6	92.0
**Phase 4**	1	0.3	2.1	99	97.9	99.7
**Phase 5**	13	9.7	15.8	87	84.2	90.3

Table S24: Phase-wise distribution (%) of classified and unclassified Robson groups with 95% confidence intervals in Gaibandha DH

Time Frame	Unclassified			Classified		
	Percentage	LCL	UCL	Percentage	LCL	UCL
**Phase 2**	31	28.9	33.0	69	67.0	71.1
**Phase 3**	30	26.4	34.2	70	65.8	73.6
**Phase 4**	5	4.0	6.4	95	93.6	96.0
**Phase 5**	1	0.5	1.6	99	98.4	99.5

Table S25: Phase-wise distribution (%) of classified and unclassified Robson groups with 95% confidence intervals in Netrokona DH

Time Frame	Unclassified			Classified		
	Percentage	LCL	UCL	Percentage	LCL	UCL
**Phase 2**	71	69.2	73.5	29	26.5	30.8
**Phase 3**	62	58.4	66.2	38	33.8	41.6
**Phase 4**	5	4.0	6.2	95	93.8	96.0
**Phase 5**	2	1.0	2.4	98	97.6	99.0

Table S26: Phase-wise distribution (%) of classified and unclassified Robson groups with 95% confidence intervals in Sunamganj DH

Time Frame	Unclassified			Classified		
	Percentage	LCL	UCL	Percentage	LCL	UCL
**Phase 2**	23	18.9	26.8	77	73.2	81.1
**Phase 3**	14	10.3	19.5	86	80.5	89.7
**Phase 4**	9	6.9	11.1	91	88.9	93.1
**Phase 5**	4	2.7	5.7	96	94.3	97.3

Table S27: Phase-wise distribution (%) of classified and unclassified Robson groups with 95% confidence intervals in Bagerhat DH

Time Frame	Unclassified			Classified		
	Percentage	LCL	UCL	Percentage	LCL	UCL
**Phase 2**	25	22.6	27.7	75	72.3	77.4
**Phase 3**	14	10.9	16.8	86	83.2	89.1
**Phase 4**	26	23.2	28.7	74	71.3	76.8
**Phase 5**	5	3.5	6.5	95	93.5	96.5

Table S28: Phase-wise distribution (%) of classified and unclassified Robson groups with 95% confidence intervals in Bhola DH

Time Frame	Unclassified			Classified		
	Percentage	LCL	UCL	Percentage	LCL	UCL
**Phase 2**	56	53.9	59.0	44	41.0	46.1
**Phase 3**	17	13.8	19.7	83	80.3	86.2
**Phase 4**	8	6.6	9.7	92	90.3	93.4
**Phase 5**	2	1.6	3.4	98	96.6	98.4

Table S29: Phase-wise distribution (%) of classified and unclassified Robson groups with 95% confidence intervals in Khagrachari DH

	Phase 2 (Sep'21-Apr'22)						Phase 3 (May'22-July'22)						Phase 4 (Aug'22-Dec'22)						Phase 5 (Jan'23-Jun'23)					
**Group**	**Number of CS in group**	**Number of women in group**	**Group Size (%)**	**Group CS rate (%)**	**Absolute group contribution to overall CS rate (%)**	**Relative contribution of group to overall CS rate (%)**	**Number of CS in group**	**Number of women in group**	**Group Size (%)**	**Group CS rate (%)**	**Absolute group contribution to overall CS rate (%)**	**Relative contribution of group to overall CS rate (%)**	**Number of CS in group**	**Number of women in group**	**Group Size (%)**	**Group CS rate (%)**	**Absolute group contribution to overall CS rate (%)**	**Relative contribution of group to overall CS rate (%)**	**Number of CS in group**	**Number of women in group**	**Group Size (%)**	**Group CS rate (%)**	**Absolute group contribution to overall CS rate (%)**	**Relative contribution of group to overall CS rate (%)**
1	40	1873	19.8	2.1	0.4	2.0	15	842	24.3	1.8	0.4	1.9	66	2510	31.2	2.6	0.8	3.5	77	2,184	28.8	3.5	1.0	3.7
2a	37	85	0.9	43.5	0.4	1.8	15	39	1.1	38.5	0.4	1.9	15	35	0.4	42.9	0.2	0.8	22	75	1.0	29.3	0.3	1.1
2b	237	237	2.5	100.0	2.5	11.6	143	143	4.1	100.0	4.1	17.7	436	436	5.4	100.0	5.4	22.8	508	508	6.7	100.0	6.7	24.4
3	26	1679	17.8	1.5	0.3	1.3	10	770	22.2	1.3	0.3	1.2	26	2279	28.3	1.1	0.3	1.4	32	2,411	31.8	1.3	0.4	1.5
4a	8	35	0.4	22.9	0.1	0.4	10	26	0.7	38.5	0.3	1.2	13	30	0.4	43.3	0.2	0.7	22	50	0.7	44.0	0.3	1.1
4b	90	90	1.0	100.0	1.0	4.4	55	55	1.6	100.0	1.6	6.8	197	197	2.4	100.0	2.4	10.3	237	237	3.1	100.0	3.1	11.4
5	464	485	5.1	95.7	4.9	22.6	247	257	7.4	96.1	7.1	30.6	821	876	10.9	93.7	10.2	43.0	923	976	12.9	94.6	12.2	44.4
6	13	44	0.5	29.5	0.1	0.6	6	25	0.7	24.0	0.2	0.7	27	72	0.9	37.5	0.3	1.4	18	65	0.9	27.7	0.2	0.9
7	23	67	0.7	34.3	0.2	1.1	7	32	0.9	21.9	0.2	0.9	49	138	1.7	35.5	0.6	2.6	45	115	1.5	39.1	0.6	2.2
8	10	68	0.7	14.7	0.1	0.5	8	35	1.0	22.9	0.2	1.0	16	135	1.7	11.9	0.2	0.8	18	134	1.8	13.4	0.2	0.9
9	5	5	0.1	100.0	0.1	0.2	0	0	0.0	-	0.0	0.0	1	1	0.0	100.0	0.0	0.1	10	10	0.1	100.0	0.1	0.5

10	66	537	5.7	12.3	0.7	3.2	42	249	7.2	16.9	1.2	5.2	102	700	8.7	14.6	1.3	5.3	116	610	8.0	19.0	1.5	5.6
Unclassified	1031	4248	44.9	24.3	10.9	50.3	250	996	28.7	25.1	7.2	30.9	142	643	8.0	22.1	1.8	7.4	53	214	2.8	24.8	0.7	2.5
Total	2050	9453	100.0	21.7	21.7	100.0	808	3469	100.0	23.3	23.3	100.0	1911	8052	100.0	23.7	23.7	100.0	2081	7589	100.0	27.4	27.4	100.0

Table S30: Robson TGCS report table overall (8 DHs) by phase

	**Phase 2 (Sep'21-Apr'22)**						**Phase 3 (May'22-July'22)**						**Phase 4 (Aug'22-Dec'22)**						**Phase 5 (Jan'23-Jun'23)**					
**Group**	**Number of CS in group**	**Number of women in group**	**Group Size (%)**	**Group CS rate (%)**	**Absolute group contribution to overall CS rate (%)**	**Relative contribution of group to overall CS rate (%)**	**Number of CS in group**	**Number of women in group**	**Group Size (%)**	**Group CS rate (%)**	**Absolute group contribution to overall CS rate (%)**	**Relative contribution of group to overall CS rate (%)**	**Number of CS in group**	**Number of women in group**	**Group Size (%)**	**Group CS rate (%)**	**Absolute group contribution to overall CS rate (%)**	**Relative contribution of group to overall CS rate (%)**	**Number of CS in group**	**Number of women in group**	**Group Size (%)**	**Group CS rate (%)**	**Absolute group contribution to overall CS rate (%)**	**Relative contribution of group to overall CS rate (%)**
1	2	121	18.4	1.7	0.3	0.9	2	45	18.9	4.4	0.8	2.7	9	137	25.7	6.6	1.7	5.0	15	114	23.5	13.2	3.1	7.8
2a	0	4	0.6	0.0	0.0	0.0	0	0	0.0	-	0.0	0.0	1	3	0.6	33.3	0.2	0.6	2	2	0.4	100.0	0.4	1.0
2b	23	23	3.5	100.0	3.5	9.9	15	15	6.3	100.0	6.3	20.3	41	41	7.7	100.0	7.7	22.9	19	19	3.9	100.0	3.9	9.9
3	1	94	14.3	1.1	0.2	0.4	0	63	26.5	0.0	0.0	0.0	5	171	32.0	2.9	0.9	2.8	4	167	34.4	2.4	0.8	2.1
4a	0	2	0.3	0.0	0.0	0.0	0	1	0.4	0.0	0.0	0.0	0	0	0.0	-	0.0	0.0	0	0	0.0	-	0.0	0.0
4b	11	11	1.7	100.0	1.7	4.7	5	5	2.1	100.0	2.1	6.8	12	12	2.2	100.0	2.2	6.7	5	5	1.0	100.0	1.0	2.6
5	92	102	15.5	90.2	14.0	39.7	38	41	17.2	92.7	16.0	51.4	100	105	19.7	95.2	18.7	55.9	135	137	28.2	98.5	27.8	70.3
6	3	4	0.6	75.0	0.5	1.3	1	1	0.4	100.0	0.4	1.4	0	2	0.4	0.0	0.0	0.0	1	3	0.6	33.3	0.2	0.5
7	6	8	1.2	75.0	0.9	2.6	2	4	1.7	50.0	0.8	2.7	3	9	1.7	33.3	0.6	1.7	5	5	1.0	100.0	1.0	2.6
8	3	5	0.8	60.0	0.5	1.3	1	8	3.4	12.5	0.4	1.4	2	10	1.9	20.0	0.4	1.1	3	9	1.9	33.3	0.6	1.6

9	0	0	0.0	-	0.0	0.0	0	0	0.0	-	0.0	0.0	0	0	0.0	-	0.0	0.0	1	1	0.2	100.0	0.2	0.5
10	4	27	4.1	14.8	0.6	1.7	3	16	6.7	18.8	1.3	4.1	4	23	4.3	17.4	0.7	2.2	1	18	3.7	5.6	0.2	0.5
Unclassified	87	257	39.1	33.9	13.2	37.5	7	39	16.4	17.9	2.9	9.5	2	21	3.9	9.5	0.4	1.1	1	5	1.0	20.0	0.2	0.5
Total	232	658	100.0	35.3	35.3	100.0	74	238	100.0	31.1	31.1	100.0	179	534	100.0	33.5	33.5	100.0	192	485	100.0	39.6	39.6	100.0

Table S31: Robson TGCS report table for Munshiganj DH (by phase)

	Phase 2 (Sep'21-Apr'22)						Phase 3 (May'22-July'22)						Phase 4 (Aug'22-Dec'22)						Phase 5 (Jan'23-Jun'23)					
**Group**	**Number of CS in group**	**Number of women in group**	**Group Size (%)**	**Group CS rate (%)**	**Absolute group contribution to overall CS rate (%)**	**Relative contribution of group to overall CS rate (%)**	**Number of CS in group**	**Number of women in group**	**Group Size (%)**	**Group CS rate (%)**	**Absolute group contribution to overall CS rate (%)**	**Relative contribution of group to overall CS rate (%)**	**Number of CS in group**	**Number of women in group**	**Group Size (%)**	**Group CS rate (%)**	**Absolute group contribution to overall CS rate (%)**	**Relative contribution of group to overall CS rate (%)**	**Number of CS in group**	**Number of women in group**	**Group Size (%)**	**Group CS rate (%)**	**Absolute group contribution to overall CS rate (%)**	**Relative contribution of group to overall CS rate (%)**
1	4	63	5.3	6.3	0.3	0.5	1	40	10.9	2.5	0.3	0.4	20	143	15.5	14.0	2.2	3.5	22	146	15.1	15.1	2.3	3.3
2a	0	1	0.1	0.0	0.0	0.0	0	1	0.3	0.0	0.0	0.0	2	9	1.0	22.2	0.2	0.3	1	3	0.3	33.3	0.1	0.1
2b	79	79	6.6	100.0	6.6	9.7	51	51	13.9	100.0	13.9	20.5	142	142	15.4	100.0	15.4	24.8	176	176	18.1	100.0	18.1	26.2
3	0	17	1.4	0.0	0.0	0.0	0	3	0.8	0.0	0.0	0.0	8	164	17.8	4.9	0.9	1.4	8	150	15.5	5.3	0.8	1.2
4a	0	0	0.0	-	0.0	0.0	0	0	0.0	-	0.0	0.0	2	8	0.9	25.0	0.2	0.3	0	1	0.1	0.0	0.0	0.0
4b	12	12	1.0	100.0	1.0	1.5	2	2	0.5	100.0	0.5	0.8	45	45	4.9	100.0	4.9	7.9	60	60	6.2	100.0	6.2	8.9
5	156	157	13.1	99.4	13.0	19.2	92	92	25.0	100.0	25.0	36.9	267	274	29.8	97.4	29.0	46.6	321	324	33.4	99.1	33.1	47.7
6	5	5	0.4	100.0	0.4	0.6	1	2	0.5	50.0	0.3	0.4	11	15	1.6	73.3	1.2	1.9	6	7	0.7	85.7	0.6	0.9
7	9	9	0.8	100.0	0.8	1.1	0	0	0.0	-	0.0	0.0	18	23	2.5	78.3	2.0	3.1	17	20	2.1	85.0	1.8	2.5
8	2	3	0.3	66.7	0.2	0.2	0	1	0.3	0.0	0.0	0.0	6	6	0.7	100.0	0.7	1.0	5	6	0.6	83.3	0.5	0.7

9	5	5	0.4	100.0	0.4	0.6	0	0	0.0	-	0.0	0.0	0	0	0.0	-	0.0	0.0	4	4	0.4	100.0	0.4	0.6
10	30	47	3.9	63.8	2.5	3.7	10	18	4.9	55.6	2.7	4.0	34	61	6.6	55.7	3.7	5.9	44	60	6.2	73.3	4.5	6.5
Unclassified	512	802	66.8	63.8	42.7	62.9	92	158	42.9	58.2	25.0	36.9	18	30	3.3	60.0	2.0	3.1	9	13	1.3	69.2	0.9	1.3
Total	814	1200	100.0	67.8	67.8	100.0	249	368	100.0	67.7	67.7	100.0	573	920	100.0	62.3	62.3	100.0	673	970	100.0	69.4	69.4	100.0

Table S32: Robson TGCS report table for Bogura DH (by phase)

	Phase 2 (Sep’21-Apr’22)						Phase 3 (May’22-July’22)						Phase 4 (Aug’22-Dec’22)						Phase 5 (Jan’23-Jun’23)					
**Group**	**Number of CS in group**	**Number of women in group**	**Group Size (%)**	**Group CS rate (%)**	**Absolute group contribution to overall CS rate (%)**	**Relative contribution of group to overall CS rate (%)**	**Number of CS in group**	**Number of women in group**	**Group Size (%)**	**Group CS rate (%)**	**Absolute group contribution to overall CS rate (%)**	**Relative contribution of group to overall CS rate (%)**	**Number of CS in group**	**Number of women in group**	**Group Size (%)**	**Group CS rate (%)**	**Absolute group contribution to overall CS rate (%)**	**Relative contribution of group to overall CS rate (%)**	**Number of CS in group**	**Number of women in group**	**Group Size (%)**	**Group CS rate (%)**	**Absolute group contribution to overall CS rate (%)**	**Relative contribution of group to overall CS rate (%)**
1	10	188	24.2	5.3	1.3	6.2	6	45	18.1	13.3	2.4	6.9	23	134	24.8	17.2	4.3	12.8	14	104	21.0	13.5	2.8	8.7
2a	3	5	0.6	60.0	0.4	1.9	1	1	0.4	100.0	0.4	1.1	0	1	0.2	0.0	0.0	0.0	0	2	0.4	0.0	0.0	0.0
2b	41	41	5.3	100.0	5.3	25.3	22	22	8.9	100.0	8.9	25.3	41	41	7.6	100.0	7.6	22.8	47	47	9.5	100.0	9.5	29.2
3	9	240	30.9	3.8	1.2	5.6	4	52	21.0	7.7	1.6	4.6	5	147	27.2	3.4	0.9	2.8	4	116	23.4	3.4	0.8	2.5
4a	0	0	0.0	-	0.0	0.0	0	0	0.0	-	0.0	0.0	1	2	0.4	50.0	0.2	0.6	0	0	0.0	-	0.0	0.0
4b	16	16	2.1	100.0	2.1	9.9	10	10	4.0	100.0	4.0	11.5	21	21	3.9	100.0	3.9	11.7	19	19	3.8	100.0	3.8	11.8
5	49	53	6.8	92.5	6.3	30.2	19	19	7.7	100.0	7.7	21.8	72	72	13.3	100.0	13.3	40.0	52	52	10.5	100.0	10.5	32.3
6	1	6	0.8	16.7	0.1	0.6	1	4	1.6	25.0	0.4	1.1	1	7	1.3	14.3	0.2	0.6	1	3	0.6	33.3	0.2	0.6
7	1	14	1.8	7.1	0.1	0.6	0	3	1.2	0.0	0.0	0.0	4	10	1.8	40.0	0.7	2.2	3	9	1.8	33.3	0.6	1.9
8	2	9	1.2	22.2	0.3	1.2	2	3	1.2	66.7	0.8	2.3	0	4	0.7	0.0	0.0	0.0	3	7	1.4	42.9	0.6	1.9
9	0	0	0.0	-	0.0	0.0	0	0	0.0	-	0.0	0.0	0	0	0.0	-	0.0	0.0	0	0	0.0	-	0.0	0.0

10	12	108	13.9	11.1	1.5	7.4	11	60	24.2	18.3	4.4	12.6	12	97	17.9	12.4	2.2	6.7	12	74	14.9	16.2	2.4	7.5
Unclassified	18	96	12.4	18.8	2.3	11.1	11	29	11.7	37.9	4.4	12.6	0	5	0.9	0.0	0.0	0.0	6	62	12.5	9.7	1.2	3.7
Total	162	776	100.0	20.9	20.9	100.0	87	248	100.0	35.1	35.1	100.0	180	541	100.0	33.3	33.3	100.0	161	495	100.0	32.5	32.5	100.0

Table S33: Robson TGCS report table for Gaibandha DH (by phase)

	Phase 2 (Sep’21-Apr’22)						Phase 3 (May’22-July’22)						Phase 4 (Aug’22-Dec’22)						Phase 5 (Jan’23-Jun’23)					
Group	Number of CS in group	Number of women in group	Group Size (%)	Group CS rate (%)	Absolute group contribution to overall CS rate (%)	Relative contribution of group to overall CS rate (%)	Number of CS in group	Number of women in group	Group Size (%)	Group CS rate (%)	Absolute group contribution to overall CS rate (%)	Relative contribution of group to overall CS rate (%)	Number of CS in group	Number of women in group	Group Size (%)	Group CS rate (%)	Absolute group contribution to overall CS rate (%)	Relative contribution of group to overall CS rate (%)	Number of CS in group	Number of women in group	Group Size (%)	Group CS rate (%)	Absolute group contribution to overall CS rate (%)	Relative contribution of group to overall CS rate (%)
1	3	460	23.4	0.7	0.2	0.8	1	132	23.4	0.8	0.2	0.8	0	444	30.3	0.0	0.0	0.0	4	433	29.4	0.9	0.3	1.2
2a	5	9	0.5	55.6	0.3	1.4	0	0	0.0	-	0.0	0.0	0	0	0.0	-	0.0	0.0	0	7	0.5	0.0	0.0	0.0
2b	56	56	2.8	100.0	2.8	15.1	22	22	3.9	100.0	3.9	18.2	97	97	6.6	100.0	6.6	29.8	96	96	6.5	100.0	6.5	28.3
3	1	494	25.1	0.2	0.1	0.3	0	124	22.0	0.0	0.0	0.0	0	426	29.1	0.0	0.0	0.0	3	470	31.9	0.6	0.2	0.9
4a	0	1	0.1	0.0	0.0	0.0	0	0	0.0	-	0.0	0.0	0	0	0.0	-	0.0	0.0	0	3	0.2	0.0	0.0	0.0
4b	40	40	2.0	100.0	2.0	10.8	14	14	2.5	100.0	2.5	11.6	47	47	3.2	100.0	3.2	14.4	58	58	3.9	100.0	3.9	17.1
5	114	117	6.0	97.4	5.8	30.8	41	43	7.6	95.3	7.3	33.9	129	134	9.1	96.3	8.8	39.6	149	158	10.7	94.3	10.1	44.0
6	1	7	0.4	14.3	0.1	0.3	1	2	0.4	50.0	0.2	0.8	1	9	0.6	11.1	0.1	0.3	1	14	1.0	7.1	0.1	0.3
7	1	8	0.4	12.5	0.1	0.3	1	6	1.1	16.7	0.2	0.8	3	22	1.5	13.6	0.2	0.9	0	17	1.2	0.0	0.0	0.0
8	0	16	0.8	0.0	0.0	0.0	0	1	0.2	0.0	0.0	0.0	0	24	1.6	0.0	0.0	0.0	3	34	2.3	8.8	0.2	0.9
9	0	0	0.0	-	0.0	0.0	0	0	0.0	-	0.0	0.0	0	0	0.0	-	0.0	0.0	0	0	0.0	-	0.0	0.0
10	13	150	7.6	8.7	0.7	3.5	5	49	8.7	10.2	0.9	4.1	20	187	12.8	10.7	1.4	6.1	22	168	11.4	13.1	1.5	6.5

Unclassified	136	608	30.9	22.4	6.9	36.8	36	170	30.2	21.2	6.4	29.8	29	75	5.1	38.7	2.0	8.9	3	14	1.0	21.4	0.2	0.9
Total	370	1966	100.0	18.8	18.8	100.0	121	563	100.0	21.5	21.5	100.0	326	1465	100.0	22.3	22.3	100.0	339	1472	100.0	23.0	23.0	100.0

Table S34: Robson TGCS report table for Netrokona DH (by phase)

	Phase 2 (Sep’21-Apr’22)						Phase 3 (May’22-July’22)						Phase 4 (Aug’22-Dec’22)						Phase 5 (Jan’23-Jun’23)					
**Group**	**Number of CS in group**	**Number of women in group**	**Group Size (%)**	**Group CS rate (%)**	**Absolute group contribution to overall CS rate (%)**	**Relative contribution of group to overall CS rate (%)**	**Number of CS in group**	**Number of women in group**	**Group Size (%)**	**Group CS rate (%)**	**Absolute group contribution to overall CS rate (%)**	**Relative contribution of group to overall CS rate (%)**	**Number of CS in group**	**Number of women in group**	**Group Size (%)**	**Group CS rate (%)**	**Absolute group contribution to overall CS rate (%)**	**Relative contribution of group to overall CS rate (%)**	**Number of CS in group**	**Number of women in group**	**Group Size (%)**	**Group CS rate (%)**	**Absolute group contribution to overall CS rate (%)**	**Relative contribution of group to overall CS rate (%)**
1	4	277	16.0	1.4	0.2	2.9	1	103	16.8	1.0	0.2	1.5	5	556	36.3	0.9	0.3	2.2	12	452	32.0	2.7	0.9	4.2
2a	0	2	0.1	0.0	0.0	0.0	1	3	0.5	33.3	0.2	1.5	1	3	0.2	33.3	0.1	0.4	2	15	1.1	13.3	0.1	0.7
2b	6	6	0.3	100.0	0.3	4.3	7	7	1.1	100.0	1.1	10.3	52	52	3.4	100.0	3.4	23.1	67	67	4.7	100.0	4.7	23.5
3	2	99	5.7	2.0	0.1	1.4	0	73	11.9	0.0	0.0	0.0	1	490	32.0	0.2	0.1	0.4	6	472	33.5	1.3	0.4	2.1
4a	0	0	0.0	-	0.0	0.0	0	0	0.0	-	0.0	0.0	0	4	0.3	0.0	0.0	0.0	5	13	0.9	38.5	0.4	1.8
4b	0	0	0.0	-	0.0	0.0	5	5	0.8	100.0	0.8	7.4	27	27	1.8	100.0	1.8	12.0	41	41	2.9	100.0	2.9	14.4
5	14	16	0.9	87.5	0.8	10.1	10	11	1.8	90.9	1.6	14.7	105	128	8.4	82.0	6.9	46.7	127	150	10.6	84.7	9.0	44.6
6	1	9	0.5	11.1	0.1	0.7	0	3	0.5	0.0	0.0	0.0	6	23	1.5	26.1	0.4	2.7	3	19	1.3	15.8	0.2	1.1
7	0	8	0.5	0.0	0.0	0.0	0	2	0.3	0.0	0.0	0.0	3	23	1.5	13.0	0.2	1.3	3	27	1.9	11.1	0.2	1.1
8	0	18	1.0	0.0	0.0	0.0	0	4	0.7	0.0	0.0	0.0	3	46	3.0	6.5	0.2	1.3	2	39	2.8	5.1	0.1	0.7
9	0	0	0.0	-	0.0	0.0	0	0	0.0	-	0.0	0.0	0	0	0.0	-	0.0	0.0	1	1	0.1	100.0	0.1	0.4

10	0	62	3.6	0.0	0.0	0.0	1	20	3.3	5.0	0.2	1.5	4	103	6.7	3.9	0.3	1.8	9	93	6.6	9.7	0.6	3.2
Unclassified	111	1239	71.4	9.0	6.4	80.4	43	383	62.4	11.2	7.0	63.2	18	77	5.0	23.4	1.2	8.0	7	22	1.6	31.8	0.5	2.5
Total	138	1736	100.0	7.9	7.9	100.0	68	614	100.0	11.1	11.1	100.0	225	1532	100.0	14.7	14.7	100.0	285	1411	100.0	20.2	20.2	100.0

Table S35: Robson TGCS report table for Sunamganj DH (by phase)

	Phase 2 (Sep’21-Apr’22)						Phase 3 (May’22-July’22)						Phase 4 (Aug’22-Dec’22)						Phase 5 (Jan’23-Jun’23)					
**Group**	**Number of CS in group**	**Number of women in group**	**Group Size (%)**	**Group CS rate (%)**	**Absolute group contribution to overall CS rate (%)**	**Relative contribution of group to overall CS rate (%)**	**Number of CS in group**	**Number of women in group**	**Group Size (%)**	**Group CS rate (%)**	**Absolute group contribution to overall CS rate (%)**	**Relative contribution of group to overall CS rate (%)**	**Number of CS in group**	**Number of women in group**	**Group Size (%)**	**Group CS rate (%)**	**Absolute group contribution to overall CS rate (%)**	**Relative contribution of group to overall CS rate (%)**	**Number of CS in group**	**Number of women in group**	**Group Size (%)**	**Group CS rate (%)**	**Absolute group contribution to overall CS rate (%)**	**Relative contribution of group to overall CS rate (%)**
1	0	123	26.8	0.0	0.0	0.0	1	68	28.1	1.5	0.4	1.5	0	243	32.7	0.0	0.0	0.0	1	219	31.1	0.5	0.1	0.6
2a	0	1	0.2	0.0	0.0	0.0	0	12	5.0	0.0	0.0	0.0	0	1	0.1	0.0	0.0	0.0	0	1	0.1	0.0	0.0	0.0
2b	6	6	1.3	100.0	1.3	22.2	10	10	4.1	100.0	4.1	15.4	12	12	1.6	100.0	1.6	7.8	37	37	5.2	100.0	5.2	21.8
3	1	138	30.1	0.7	0.2	3.7	0	60	24.8	0.0	0.0	0.0	0	215	28.9	0.0	0.0	0.0	1	212	30.1	0.5	0.1	0.6
4a	1	10	2.2	10.0	0.2	3.7	0	5	2.1	0.0	0.0	0.0	0	0	0.0	-	0.0	0.0	0	0	0.0	-	0.0	0.0
4b	4	4	0.9	100.0	0.9	14.8	6	6	2.5	100.0	2.5	9.2	10	10	1.3	100.0	1.3	6.5	18	18	2.6	100.0	2.6	10.6
5	4	4	0.9	100.0	0.9	14.8	23	23	9.5	100.0	9.5	35.4	65	77	10.3	84.4	8.7	42.5	74	82	11.6	90.2	10.5	43.5
6	1	1	0.2	100.0	0.2	3.7	2	2	0.8	100.0	0.8	3.1	1	4	0.5	25.0	0.1	0.7	2	6	0.9	33.3	0.3	1.2
7	0	1	0.2	0.0	0.0	0.0	2	2	0.8	100.0	0.8	3.1	3	5	0.7	60.0	0.4	2.0	0	3	0.4	0.0	0.0	0.0
8	1	2	0.4	50.0	0.2	3.7	0	1	0.4	0.0	0.0	0.0	2	5	0.7	40.0	0.3	1.3	0	6	0.9	0.0	0.0	0.0

9	0	0	0.0	-	0.0	0.0	0	0	0.0	-	0.0	0.0	0	0	0.0	-	0.0	0.0	1	1	0.1	100.0	0.1	0.6
10	4	65	14.2	6.2	0.9	14.8	7	18	7.4	38.9	2.9	10.8	20	106	14.2	18.9	2.7	13.1	19	92	13.0	20.7	2.7	11.2
Unclassified	5	104	22.7	4.8	1.1	18.5	14	35	14.5	40.0	5.8	21.5	40	66	8.9	60.6	5.4	26.1	17	28	4.0	60.7	2.4	10.0
Total	27	459	100.0	5.9	5.9	100.0	65	242	100.0	26.9	26.9	100.0	153	744	100.0	20.6	20.6	100.0	170	705	100.0	24.1	24.1	100.0

Table S36: Robson TGCS report table for Bagerhat DH (by phase)

	Phase 2 (Sep'21-Apr'22)						Phase 3 (May'22-July'22)						Phase 4 (Aug'22-Dec'22)						Phase 5 (Jan'23-Jun'23)					
**Group**	**Number of CS in group**	**Number of women in group**	**Group Size (%)**	**Group CS rate (%)**	**Absolute group contribution to overall CS rate (%)**	**Relative contribution of group to overall CS rate (%)**	**Number of CS in group**	**Number of women in group**	**Group Size (%)**	**Group CS rate (%)**	**Absolute group contribution to overall CS rate (%)**	**Relative contribution of group to overall CS rate (%)**	**Number of CS in group**	**Number of women in group**	**Group Size (%)**	**Group CS rate (%)**	**Absolute group contribution to overall CS rate (%)**	**Relative contribution of group to overall CS rate (%)**	**Number of CS in group**	**Number of women in group**	**Group Size (%)**	**Group CS rate (%)**	**Absolute group contribution to overall CS rate (%)**	**Relative contribution of group to overall CS rate (%)**
1	1	377	33.0	0.3	0.1	1.6	2	208	37.8	1.0	0.4	11.1	0	393	38.4	0.0	0.0	0.0	0	331	38.0	0.0	0.0	0.0
2a	0	0	0.0	-	0.0	0.0	1	3	0.5	33.3	0.2	5.6	0	0	0.0	-	0.0	0.0	0	0	0.0	-	0.0	0.0
2b	3	3	0.3	100.0	0.3	4.8	0	0	0.0	-	0.0	0.0	4	4	0.4	100.0	0.4	10.3	9	9	1.0	100.0	1.0	26.5
3	1	397	34.7	0.3	0.1	1.6	2	211	38.4	0.9	0.4	11.1	0	222	21.7	0.0	0.0	0.0	0	397	45.5	0.0	0.0	0.0
4a	0	0	0.0	-	0.0	0.0	1	2	0.4	50.0	0.2	5.6	0	1	0.1	0.0	0.0	0.0	0	0	0.0	-	0.0	0.0
4b	1	1	0.1	100.0	0.1	1.6	0	0	0.0	-	0.0	0.0	0	0	0.0	-	0.0	0.0	4	4	0.5	100.0	0.5	11.8
5	9	10	0.9	90.0	0.8	14.3	5	7	1.3	71.4	0.9	27.8	24	24	2.3	100.0	2.3	61.5	15	18	2.1	83.3	1.7	44.1
6	0	4	0.3	0.0	0.0	0.0	0	8	1.5	0.0	0.0	0.0	0	3	0.3	0.0	0.0	0.0	0	8	0.9	0.0	0.0	0.0
7	1	10	0.9	10.0	0.1	1.6	2	8	1.5	25.0	0.4	11.1	3	14	1.4	21.4	0.3	7.7	3	9	1.0	33.3	0.3	8.8
8	0	6	0.5	0.0	0.0	0.0	0	3	0.5	0.0	0.0	0.0	0	28	2.7	0.0	0.0	0.0	0	15	1.7	0.0	0.0	0.0
9	0	0	0.0	-	0.0	0.0	0	0	0.0	-	0.0	0.0	1	1	0.1	100.0	0.1	2.6	0	0	0.0	-	0.0	0.0

10	0	49	4.3	0.0	0.0	0.0	0	25	4.5	0.0	0.0	0.0	1	69	6.7	1.4	0.1	2.6	2	39	4.5	5.1	0.2	5.9
Unclassified	47	287	25.1	16.4	4.1	74.6	5	75	13.6	6.7	0.9	27.8	6	265	25.9	2.3	0.6	15.4	1	42	4.8	2.4	0.1	2.9
Total	63	1144	100.0	5.5	5.5	100.0	18	550	100.0	3.3	3.3	100.0	39	1024	100.0	3.8	3.8	100.0	34	872	100.0	3.9	3.9	100.0

Table S37: Robson TGCS report table for Bhola DH (by phase)

	Phase 2 (Sep'21-Apr'22)						Phase 3 (May'22-July'22)						Phase 4 (Aug'22-Dec'22)						Phase 5 (Jan'23-Jun'23)					
**Group**	**Number of CS in group**	**Number of women in group**	**Group Size (%)**	**Group CS rate (%)**	**Absolute group contribution to overall CS rate (%)**	**Relative contribution of group to overall CS rate (%)**	**Number of CS in group**	**Number of women in group**	**Group Size (%)**	**Group CS rate (%)**	**Absolute group contribution to overall CS rate (%)**	**Relative contribution of group to overall CS rate (%)**	**Number of CS in group**	**Number of women in group**	**Group Size (%)**	**Group CS rate (%)**	**Absolute group contribution to overall CS rate (%)**	**Relative contribution of group to overall CS rate (%)**	**Number of CS in group**	**Number of women in group**	**Group Size (%)**	**Group CS rate (%)**	**Absolute group contribution to overall CS rate (%)**	**Relative contribution of group to overall CS rate (%)**
1	16	264	17.4	6.1	1.1	6.6	1	201	31.1	0.5	0.2	0.8	9	460	35.6	2.0	0.7	3.8	9	385	32.7	2.3	0.8	4.0
2a	29	63	4.2	46.0	1.9	11.9	12	19	2.9	63.2	1.9	9.5	11	18	1.4	61.1	0.9	4.7	17	45	3.8	37.8	1.4	7.5
2b	23	23	1.5	100.0	1.5	9.4	16	16	2.5	100.0	2.5	12.7	47	47	3.6	100.0	3.6	19.9	57	57	4.8	100.0	4.8	25.1
3	11	200	13.2	5.5	0.7	4.5	4	184	28.5	2.2	0.6	3.2	7	444	34.4	1.6	0.5	3.0	6	427	36.2	1.4	0.5	2.6
4a	7	22	1.5	31.8	0.5	2.9	9	18	2.8	50.0	1.4	7.1	10	15	1.2	66.7	0.8	4.2	17	33	2.8	51.5	1.4	7.5
4b	6	6	0.4	100.0	0.4	2.5	13	13	2.0	100.0	2.0	10.3	35	35	2.7	100.0	2.7	14.8	32	32	2.7	100.0	2.7	14.1
5	26	26	1.7	100.0	1.7	10.7	19	21	3.3	90.5	2.9	15.1	59	62	4.8	95.2	4.6	25.0	50	55	4.7	90.9	4.2	22.0
6	1	8	0.5	12.5	0.1	0.4	0	3	0.5	0.0	0.0	0.0	7	9	0.7	77.8	0.5	3.0	4	5	0.4	80.0	0.3	1.8
7	5	9	0.6	55.6	0.3	2.0	0	7	1.1	0.0	0.0	0.0	12	32	2.5	37.5	0.9	5.1	14	25	2.1	56.0	1.2	6.2
8	2	9	0.6	22.2	0.1	0.8	5	14	2.2	35.7	0.8	4.0	3	12	0.9	25.0	0.2	1.3	2	18	1.5	11.1	0.2	0.9
9	0	0	0.0	-	0.0	0.0	0	0	0.0	-	0.0	0.0	0	0	0.0	-	0.0	0.0	3	3	0.3	100.0	0.3	1.3

10	3	29	1.9	10.3	0.2	1.2	5	43	6.7	11.6	0.8	4.0	7	54	4.2	13.0	0.5	3.0	7	66	5.6	10.6	0.6	3.1
Unclassified	115	855	56.5	13.5	7.6	47.1	42	107	16.6	39.3	6.5	33.3	29	104	8.0	27.9	2.2	12.3	9	28	2.4	32.1	0.8	4.0
Total	244	1514	100.0	16.1	16.1	100.0	126	646	100.0	19.5	19.5	100.0	236	1292	100.0	18.3	18.3	100.0	227	1179	100.0	19.3	19.3	100.0

Table S38: Robson TGCS report table for Khagrachari DH (by phase)

Reference:

1. Ministry of Health and Family Welfare GoB. National Health Information System, District Health Information System (DHIS, version 2) [cited 2021]. Available from: https://centraldhis.mohfw.gov.bd/dhismohfw/dhis-web-commons/security/login.action.

2. Ministry of Health and Family Welfare GoB. Directorate General of Health Services (DGHS), Real-Time Health Information Dashboard [cited 2021]. Available from: https://dashboard.dghs.gov.bd/pages/hrm_post_summary.php.
